# Mass drug administration approved and candidate anthelmintics beginning on larval stage 1 *Caenorhabditis elegans* are generally potent, suggesting a novel, pre-infective control for helminths

**DOI:** 10.1371/journal.pone.0346795

**Published:** 2026-04-17

**Authors:** Sam A. Attalla, Kathryn J. Isaac, Morgan Pfeffer, Justin R. Hockaday, Raegan Weatherly, Malia B. Asselin, Allison K. Lewis, Viveke Rai, William A. Huff Jr, Mason H. Long, Tashna Placide, Kala R. Pearce, Leopold N. Nkengbeza, Anna K. Thurman, Audrey L. Cox, Giselle Domingo Diaz, Jasmine Carter, Nathan Stein, Sam Fischer, Brian L. Ellis

**Affiliations:** 1 Department of Biology, Lipscomb University, 1 University Park Dr., Nashville, Tennessee, United States of America; 2 Department of Public Health Sciences, Data Science Lab, Fred Hutch Cancer Center, Seattle, Washington, United States of America; North-Eastern Hill University, India, INDIA

## Abstract

About 1.5 billion people are infected with at least one of three soil-transmitted helminths (STHs) — roundworm, hookworm, and whipworm— which have devastating outcomes for growth, nutrition, cognition, and school attendance, trapping them in poverty. The WHO currently approves only two anthelmintics with only one mechanism of action for mass drug administration (MDA), and resistance has been documented. This calls for new drugs and new strategies to reduce worm burden. Previous research, beginning with larval stage 4 (L4) *C. elegans* as a model, used a health-rating system to score individual worms in varying concentrations of anthelmintics. We hypothesized that younger worms would be more susceptible to drugs. We tested beginning at larval stage 1 (L1) *C. elegans*, using the health-rating system, with the same drugs, and additionally tested mebendazole, from both the L1 and L4 stages, allowing comparison at different stages. We found that L1 worms are susceptible to all anthelmintics tested, including the MDA drug of choice, albendazole, but surprisingly showed significantly lower efficacy with pyrantel and nitazoxanide at the L1 stage, as measured by motility and fraction alive, compared to L4. Furthermore, mortality and inhibition tended to begin earlier in the L4 stages. Finally, we found that ivermectin was the most potent anthelmintic against L1, as previously shown for L4. Beyond providing systematic drug-to-drug and stage-to-stage comparisons that can guide therapeutic development for larval-stage helminth infections, our findings suggest, for the first time, the possibility of spraying the environment with anthelmintics to reduce hookworm populations before people become infected. As hookworm is not infective until the L3 stage from soil, targeting L1-L3 larvae presents a strategic intervention window. Because previous work on C. elegans showed lower benzimidazole drug susceptibility than helminths, our L1 results may represent conservative estimates of efficacy against STH larvae *in vitro* and in environmental applications.

## Introduction

Nearly a quarter of the global population, ~ 1.5 billion people, currently suffer from an infection from one or more soil-transmitted helminths (STH). Because of the nature of transmission being contamination and poor sanitation, these infections disproportionately affect poor areas—specifically burdening children and pregnant women (and their unborn child), which accounts for over 77% of the total infected population [1].

STHs are a group of intestinal parasitic nematodes that can infect humans through contact with larvae in contaminated soil or by ingesting eggs from contaminated water or food. They are considered a Neglected Tropical Disease (NTD). The three most prolific in this group of nematodes consist of *Ascaris lumbricoides*, *Trichuris trichiura* (whipworm), and five species of hookworm: *Necator americanus*, *Ancylostoma duodenale*, *Ancylostoma ceylanicum, Ancylostoma caninum,* and *Ancylostoma braziliense*. For *Ascaris*, the transmission route is fecal-oral, during which a person likely comes into contact with contaminated food or water, thus ingesting the eggs. The eggs hatch into larval stage 1 (L1) worms, which progressively grow into larval stage 4 (L4) worms as they migrate through the intestinal mucosa, travel through the bloodstream to the lungs, are coughed up, and swallowed back down to the small intestine, where the adult worms reside. Whipworms are also transmitted via the oral-fecal route, following a similar lifecycle progression; however, they do not enter the pulmonary circulation. Instead, they attach to the intestinal villi and take up residence in the ascending colon and cecum. In contrast, typical hookworm infection is transmitted through direct contact with the L3 infectious stage larvae in contaminated soil. After penetrating the skin, the larvae travel through the pulmonary circulation, migrate to the lungs, penetrate the pulmonary alveoli, are coughed up, and swallowed. The larvae progress to the small intestine and develop into adult, egg-laying worms. Without medication, these parasites can survive for months to years in the human intestinal tract [2].

Once infected, the adult worms reside in the human intestinal tract and continue to produce thousands of eggs per day while feeding on host tissue and/or nutrients. Those infected by STHs experience a variety of symptoms, including diarrhea, abdominal pain, intestinal blood loss, malnutrition, loss of appetite, general malaise and weakness, as well as a stunting of cognitive and physical development [3–5]. These symptoms can cause people to get stuck in a cycle of poverty or a “parasite trap” during which those already living in poverty are more likely to become infected due to unsanitary living conditions, resulting in malaise and other factors leading to a decrease in work efficiency and/or income, thus forcing them deeper into poverty and recurrent infection. While severe infections can result in intestinal blockages and require immediate surgical intervention, typical non-invasive treatment includes both proactive and reactive treatment with approved anthelmintic drugs. The WHO currently approves only two benzimidazoles for MDA—albendazole and mebendazole—having previously also approved nicotinic acetylcholine receptor agonists (pyrantel and levamisole) [6]. While all four drugs are safe and effective [7,8], they use only two mechanisms of action. The benzimidazoles disrupt parasite metabolism by inhibiting β-tubulin polymerization during microtubule assembly, thereby preventing nutrient uptake and leading to cell death [9]. The nicotinic acetylcholine receptor (nAChR) agonists act as open channel blockers for the acetylcholine receptors, thus depolarizing neuromuscular junctions, resulting in the irreversible spastic paralysis of the nematodes, causing them to detach from the intestinal walls and be expelled through the bowels [9,10]. Due to the lack of variability in these mechanisms of action and the widespread, continuous use, resistance has been observed among all four therapeutic agent, underscoring the need for the development of novel pharmaceuticals that maintain the safety and efficacy outlined above [11–13].

Prolonged reliance on current treatments will only worsen the observed resistance. Continuing to ignore this problem and persisting with the current drugs will require higher doses of each drug to reach appropriate cure rates—further hastening the development of anthelmintic resistance. While the lack of standardization does raise concerns about the generalizability of the current data, there is still evidence to show that the current anthelmintics are no longer yielding preferable cure rates; that, in conjunction with the undeniable anthelmintic resistance observed among livestock [14–17], ought to be enough to call for the research of novel anthelmintics. The candidate MDA anthelmintic drugs tested in this study include nitazoxanide (NTZ) and ivermectin (IVM), pyrantel (PYR) (formerly approved for MDA), while the currently MDA-approved medications included in the study were albendazole (ALB) and mebendazole (MEB). It is known that ivermectin primarily targets the avr-14 group of subunits that comprise glutamate-gated chloride channels, irreversibly opening the channels [18,19]. This causes an inhibition of several actions: pharyngeal pumping (feeding mechanism), motility, egg/microfilaria release (reproduction), and/or release of host immunosuppressants through excretory/secretory pores [19,20]—thus contributing to the relatively quick mortality of the worm. Additionally, it has long been known that ivermectin is safe for humans at relatively low doses. While the Food and Drug Administration (FDA) has deemed doses up to 200 µg/kg safe for human use, studies have shown that doses up to 10 times that amount are well tolerated [21]. The plan to utilize ivermectin in MDA has also already been included in the WHO roadmap for neglected tropical diseases, to be achieved by 2030 [6]. Nitazoxanide also targets glutamate-gated chloride channels; however, current data indicate that it may not do so in the same manner as ivermectin, but this remains unclear [22]. Additionally, at therapeutic doses, nitazoxanide is safe in children and adults [23,24].

Here, we use the free-living nematode *C*. *elegans* as a model for studying anthelmintics. Although not an STH, it is a great model that is well characterized, cost-effective, and easy to maintain. Because many anthelmintic targets and pharmacological responses are conserved across nematodes, *C. elegans* has been utilized in numerous veterinary anthelmintic studies [25]. They have also been shown to be valuable for human anthelmintic drug screening [26,27], drug targets [28], drug combinations [29], and anthelmintic metabolism/transcriptional studies [30]. Weaver *et al*., 2017 [27] used a health rating scale, first described in Hu *et al*., 2013 [31], to assess the health of L4 *C. elegans* treated with anthelmintics at varying doses over 7 days, rather than simply alive or dead, teasing out how effective the drugs really are. Hu *et al*. further showed qualitatively and quantitatively similar drug effects in helminths compared to *C. elegans*, but strikingly greater benzimidazole lethality in parasites, indicating that *C. elegans* responses provide a conservative estimate of expected efficacy [31].

This was also significant because it demonstrated that the drug of choice for MDA, albendazole, had a dose-dependent effect on *C. elegans*, even though it did not kill *C. elegans*, and because it provided a system to tease out health effects in much more detail. The health-rating system helps ensure that useful drugs that intoxicate but do not kill are not overlooked, as it is not essential for anthelmintic drugs to kill the parasites; instead, they should make the parasites unhealthy and aid the immune system in fighting them. Additional benefits of using *C. elegans* as a model for helminths include that it is much cheaper and easier to maintain, so that labs that do not have the money, facilities, or staff to work with parasites can still make a positive impact on the field. For example, they can increase the screening and study of more potential drugs as anthelmintics using *C. elegans,* and then the best candidates can be forwarded to labs that work with STHs. Therefore, a robust system for evaluating health is essential.

Here, we utilize the same scoring system, beginning with L1 *C. elegans,* testing the same doses as Weaver *et al*. [27] used (excluding 1000 μg/mL due to visibility issues) over 7 days, to be able to compare to the L4 data, in hopes of understanding the biology of drugging the worms earlier in their life cycle, which corresponds to drugging parasitic worms at earlier stages during infections, in particular in tissues, and give insight into timing and dosage. We also evaluate the efficacy of mebendazole beginning on L4 *C. elegans*. Though the benzimidazoles are the drug of choice for MDA, the Weaver paper did not perform the experiments with mebendazole, thus leaving a void in the field that needed to be filled. We predicted that younger worms would be more susceptible to drugs because their life stage is more focused on growth, and generally, drugs work better in situations of rapidly dividing cells (for example, drugs in the cancer, antibiotics, and parasite fields) [32–35] and because numerous studies have reported that organisms in the early life stages were more sensitive to toxicants than their adult counterparts [36–42]. Further, because hookworm is not infective until the L3 larval stage, determining whether anthelmintic drugs could affect earlier larval stages warrants and promotes further studies into the possibility of spraying fields and other contaminated areas with anthelmintics, rather than requiring patients to take the drugs, as a new, potentially compelling way to control STHs. Given that benzimidazoles are less lethal to *C. elegans* than to parasitic nematodes [27,31], the L1-stage effects we observe could be viewed as a conservative lower bound on expected larval susceptibility in target parasites; however, environmental efficacy will need to be determined experimentally. While spraying fields to control insects and fungus is common practice, to our knowledge, this is the first time such a control method has been proposed for the larval stages of STHs, although Partridge *et al*. mentioned the possibility of spraying the environment to control whipworm eggs [43].

## Materials and methods

### Ethics statement

Ethics approval was not required because non-regulated animals were used in this study. All experiments complied with institutional guidelines for laboratory handling of invertebrates.

### Chemicals and reagents

#### CaCl_2_ 1 M.

This solution was prepared by adding 110.99 grams of Calcium Chloride to 1 liter of dH_2_O and mixing on a stir plate until homogeneous, followed by suction filtration before storage.

#### MgSO_4_ 1 M.

This solution was prepared by adding 120.37 grams of MgSO_4_–1 liter of dH_2_O and mixing on a stir plate until homogeneous, followed by suction filtration before storage.

#### KPO_4_ 1 M (pH 6).

This solution was prepared by adding 108.3 grams of KH_2_PO_4_ and 35.6 grams of K_2_HPO_4_–1 liter of dH_2_O, followed by autoclaving before storage.

#### Cholesterol 5 mg/mL.

This solution was prepared by adding 0.25 grams of Cholesterol to 50 mL of pure ethanol (100%, 200 proof) and vortexing until dissolved, followed by 0.22 μm syringe filtration before storage.

#### KOH 5 M.

This solution was prepared by adding 280 grams of KOH to 1 liter of dH_2_O and mixing on a stir plate until homogeneous, followed by autoclaving before storage.

#### Potassium (K)-Citrate tribasic monohydrate (1 M).

This solution was prepared by adding 324.41 grams of Potassium Citrate tribasic monohydrate to 1 liter of dH_2_O and mixing on a stir plate until homogeneous, followed by autoclaving before storage.

#### S-Basal.

This solution was prepared by adding 5.85 grams of NaCl, 1 gram of K_2_HPO_4_, and 6 grams of KH_2_PO_4_–1 liter of dH_2_O before autoclaving and storing at room temperature.

#### Trace Metals Solution.

This solution was prepared in dH_2_O and consisted of 5 mM EDTA, 2.5 mM FeSO_4_, 1.0 mM MnCl_2_, 1 mM ZnSO_4_, and 0.1 mM CuSO_4_, poured into dark glass bottles and autoclaved before being stored at room temperature.

#### M9 Buffer.

This solution was prepared by adding 2.5 grams of NaCl, 0.30 grams of Na_2_HPO_4_, and 0.15 grams of KH_2_PO_4_–500 mL of dH_2_O before autoclaving. After autoclaving, 500 µL of 1M MgSO_4_ is added before storage.

#### FudR (8mM).

This solution was prepared by first dissolving 50 grams of FudR in 1 mL of sterile dH_2_O, followed by an additional 24.39 mL of sterile dH_2_O. The solution was then filter sterilized, aliquoted into bullet tubes, and stored at −20 °C.

#### S-Media.

This solution was prepared by adding 100 µL of K-Citrate (1 M), 100 µL of Trace Metals, 30 µL of MgSO_4_ (1 M), 30 µL of CaCl_2_ (1 M), and 10 µL of cholesterol (5 mg/mL in EtOH) to 10 mL of S-Basal directly before setting up an experimental assay.

#### Drug sourcing.

Most drugs were purchased from Sigma-Aldrich: albendazole (catalog no. A4673), mebendazole (catalog no. M2523), ivermectin (catalog no. I8898), and pyrantel pamoate (catalog no. P6210). Romark Laboratories kindly provided Nitazoxanide.

### *C. elegans* maintenance, chunking, and bleaching [44,45]

#### Nematode Growth (NG) Plates:.

The Nematode Growth (NG) Plates solution was prepared the night before pouring plates. This solution was made in batches, resulting in 80–100 of the 60 mm plates. The solution consisted of 972 mL of dH_2_O, 3 grams of NaCl, 2.5 grams of Bacto Peptone, and 20 grams of Bacto Agar mixed. The solution was then covered with aluminum foil and refrigerated overnight before autoclaving the next morning. After autoclaving, several other components were added to the solution in the fume hood. This included 1 mL of 5 mg/mL Cholesterol (in pure ethanol), 1 mL of 1 M CaCl_2_, 1 mL of 1 M MgSO_4_, and 25 mL of 1 M potassium phosphate (pH 6), all of which were filter-sterilized, except the potassium phosphate, which was autoclaved. The solution was mixed before being poured into 60 mm plates ~ ⅓ of the way full. The plates were allowed to solidify and dry for 1–2 days (with lids on) and finally stored upside down in the lab refrigerator.

#### Enriched Nematode Growth (ENG) Plates.

The Enriched Nematode Growth (ENG) Plates solution was prepared the night before pouring plates. This solution was made in batches, resulting in 40–60 of the 100 mm plates. The solution consisted of 972 mL of dH_2_O, 3 grams of NaCl, 5 grams of Bacto Peptone, 1 gram of Bacto yeast extract, and 20 grams of Bacto Agar mixed. The solution was then covered with aluminum foil and refrigerated overnight before autoclaving the next morning. After autoclaving, several other components were added to the solution in the fume hood. This included 1 mL of 5 mg/mL Cholesterol (in pure ethanol), 1 mL of 1 M CaCl_2_, 1 mL of 1 M MgSO_4_, and 25 mL of 1 M potassium phosphate (pH 6), all of which were filter sterilized apart from the potassium phosphate, which was autoclaved. The solution was mixed before being poured into 100 mm plates, ~ ⅓ of the way full. The plates were allowed to solidify and dry for 1–2 days (with lids on) and finally stored upside down in the lab refrigerator.

Maintenance, chunking, bleaching, and seeding were completed as done previously [45,46]. Briefly, the *C. elegans* Bristol strain, N2 ancestral, was maintained on 6 cm Nematode Growth (NG) plates with three drops of ~50 μL OP50 strain *Escherichia coli (E. coli*) of an O.D. between 0.7 and 2.0 for a food source. One or two L4 stage worms were transferred to a new NG plate using a platinum pick every three days to maintain a healthy stock of worms. For chunking, to begin the process of the experiment, an NG plate is cut into quarters and one quarter is flipped and patted down onto an Enriched Nematode Growth (ENG) 10 cm plate that has a thin layer of OP50 (~300 uL of O.D. 0.7-2.0) on top of the agar to allow for the worms to grow further and populate the larger area. For bleaching, to synchronize the *C. elegans* at the egg stage, ENG plates well populated with healthy worms were washed off the plate with dH_2_O and transferred to a Falcon tube. The tube was spun in a clinical centrifuge for 1 minute at ~5000 rpm, and the supernatant was removed until 3.5 mL remained. Next, 1 mL of bleach and 0.5 mL of 5M KOH were added. The tube was vigorously shaken by hand for 2–5 minutes, and the contents were observed under a dissecting microscope every 30 seconds until half of the worms had dissolved. Immediately following, the worms were washed twice with dH_2_O and once with M9 buffer, spinning down the egg pellet between washes in a clinical centrifuge at 5000 rpm for 1 minute. Next, the lid was turned ¼ turn open, wrapped in parafilm, and rocked overnight at room temperature so the eggs hatched but stayed in the L1 stage due to a lack of food. The L1 worms were used for liquid assays the day following the bleaching (see subsection below “*in vitro* liquid assays”). The seeding process was performed only for experiments using L4 worms and was carried out the day following bleaching, precisely 44 hours before setting up the assays. The Falcon tube containing L1 worms was lightly shaken to resuspend the worms and obtain as even a mixture as possible. Next, 400 µL per plate of the mixture was pipetted onto two new ENG plates with OP50, each. The plates were then stored in a 20-degree incubator. Precisely 44 hours after seeding, the L4 worms were collected from the plate using sterile water and utilized in the liquid assays (see below).

### *in vitro* liquid assays

Experiments were set up in 24-well polystyrene plates (non-tissue culture-treated). Each well contained 5−20 age synchronized L1 worms (or L4 worms for the L4 MBZ experiments), 10 µL of varying concentrations of drug (or 10 µL of 10% DMSO for the control wells), and 478 µL of a cocktail which contained 365 µL of S-media, 12.5 µL of 8mM 5-Fluoro-2’-deoxyuridine (FudR), and 100 µL of 3.0 OD OP50. FudR was added to inhibit egg hatching. Each of the varying drug concentrations (0.1, 1.0, 10, 100, and 1000 µg/mL) was achieved through 10-fold serial dilutions during which 10 mg of the drug was dissolved in 20 µL of 100% DMSO, followed by the addition of 180 µL of dH_2_O, resulting in the stock concentration. 10 µL of this stock solution was added to a well of the 24-well plate with 490 µL, resulting in a final concentration of 1000 µg/mL in the well. 20 µL of the stock concentration was added to 180 µL of 10% DMSO (90% dH_2_O), resulting in the next-highest drug concentration. This dilution process was repeated three more times, and 10 µL was used per well, to achieve each drug concentration for the final concentrations in wells outlined above (100, 10, 1.0, and 0.1 µg/mL). Note that the 1000 µg/mL final concentration was used only in experiments involving larval stage 4 (L4) worms, due to consistent visibility issues with L1 worms, given their size and the high drug concentration in the wells. Each well, including the control, had a final DMSO concentration of 0.2%, as in Weaver *et al*., 2017 [27].

Each drug concentration and the negative control were tested in triplicate. Each worm in every well was scored daily on a 0−3 motility rating scale for 7 days. 7 days was chosen for two reasons: one, to provide a precedent for comparison with Weaver *et al*. and Hu *et al*.; and two, because the mean lifespan of N2 worms in liquid culture at 25 °C is 9 days, meaning they begin to look sick before then [47]. Importantly, Hu *et al*. showed that the anthelmintics on helminths *in vitro* were efficacious, which, along with the data from Weaver and this manuscript, will help to guide initial controlled environmental testing. The scoring is as follows: 3 represents a worm with vigorous movement similar to control no drug; 2 represents a worm with whole-body movements (observed without external stimulus) significantly slower than control no drug; 1 represents a worm that was not moving on its own but moved when touched with a probe (an eyelash pick) (tested at three different body locations); and 0 represents a worm that did not move even when prodded at those three locations and is termed “dead” although it is possible the worm was still alive but not moving. Each experiment was performed at least 3 times, with each researcher having completed training using L4 worms treated with albendazole to replicate the experiments from Weaver *et al*., 2017, to help reduce potential bias in scoring a 3 vs a 2. To eliminate bias in determining a 3 versus a 2 (and thus completely eliminate all bias from the scoring system), a new scoring system was also created using a 2−0 scoring system (S1–S6 Figs, S1 Table). In this system, the worms scored as a 3 or a 2 in the 3−0 system were counted as a 2. The rest of the systems were the same—a 1 in the 3−0 system was also a 1 in the 2−0 system, and a 0 in the 3−0 system was a 0 in the 2−0 system. The data were consistent with the trends observed in the 3−0 system, with the obvious caveat that it was not possible to tease out a fully healthy worm from a moderately sick one. The total number of nematodes treated by drug and concentration is outlined in Table 1.

**Table 1:** Number of *C. elegans* treated.

	PYR (L1)	NTZ (L1)	IVM (L1)	ALB (L1)	MBZ (L1)	MBZ (L4)	Total (per concentration)
**0.0 µg/mL**	173	215	318	362	282	89	1,439
**0.1 µg/mL**	192	266	312	346	290	90	1,496
**1.0 µg/mL**	197	242	305	338	279	78	1,439
**10.0 µg/mL**	174	264	303	333	292	93	1,459
**100.0 µg/mL**	165	247	262	325	211	83	1,293
**1000 µg/mL**	N/A	N/A	N/A	N/A	N/A	63	63
**Total** (per drug)	901	1,234	1,500	1,704	1,354	496	**Grand Total:** 7,189

Total number of *C. elegans* treated across multiple experiments, separated by drug and concentration. N/A = not applicable because it was not performed.

### Data analyses

Individual experiments with controls that averaged less than a 2.2 motility index score on day 7 were excluded from this study. This value was chosen *a priori* based on initial data from control worms alone (data not shown). Additionally, if an experiment was missing data for a well for at least one day, the experiment was excluded.

GraphPad Prism version 10.5 (GraphPad Software Inc.) was used to perform data analyses (the same version was used throughout. GraphPad was used for transformations and plotting for all of the various analyses: motility index score and fraction alive for the length of the experiment (7 days) and averaged across experiments (n = 9 for ALB; n = 7 for PYR; n = 6 for IVM; n = 6 for NTZ; n = 9 for L1 MBZ; and n = 3 for L4 MBZ, with a minimum of 496 worms per drug and 63 worms per individual drug concentration (Table 1)), IC_50_ and LC_50_ on day 4 for pooled experiments, as well as IT_50_ and LT_50_ for one representative experiment per drug/stage combination (ALB-L1-3, PYR-L1-8, IVM-L1-3, NTZ-L1-4, MBZ-L1-10, and MBZ-L4-5).

### Scoring data transformations

All experiments used a 3-2-1-0 health rating, a motility-based system explained earlier. Scoring data transformations were performed, translating these scores into a binary alive/dead 1−0 score and then into a simplified motility-based 2-1-0 system that distinguishes healthy worms from those that require external stimulus to move or are dead (Table 2).

**Table 2:** Scoring systems for testing efficacy of anthelmintics on *C. elegans.*

Originally assigned 3-2-1-0 health rating system score	Translated binary alive/dead 1−0 scoring system	Translated simplified motility-based 2-1-0 scoring system
3	1	2
2	1	2
1	1	1
0	0	0

These scoring systems are used to evaluate the efficacy of anthelmintics and their relationships. Notice that scores are assigned daily to every *C. elegans*, at every concentration, as either 3, 2, 1, or 0, as was performed in Weaver *et al*., 2017. Then, that data was translated into the unbiased 1/0 binary alive-dead system, or the unbiased 2/1/0 health-rating system. Grayed squares indicate “inhibited” for that given scoring system.

### Motility index score line graphs

The health rating system was used to score each worm every day for 7 days. For each well in the experiment each day the motility index score of that given well was found by weighting the number of worms of each score by its assigned score and summing those weights then dividing by the total number of worms in the well (e.g.,: [(0 x number of worms scored 0) + (1 x number of worms scored 1) + (2 x number of worms scored 2) + (3 x number of worms scored 3)] / total number of worms in the well). Note that all wells were assigned a motility index score of 3 at day 0 (when worms were added to the well plate, the day before observation/scoring began). The motility index scores from related concentration wells were averaged across all relevant same-life-stage/drug-treatment experiments. If an experiment was performed n = 5 times, the average was taken across 15 wells (3 replicates per experiment x 5 experimental runs) by treating all wells as one, with the total motility score divided by the total number of worms. The resulting averages were then graphed as line graphs, with the motility index score on the y-axis, the days on the x-axis, and line color denoting the drug concentration.

### Fraction survival line graphs

The health rating system was used to score each worm daily for 7 days, and the resulting data were translated into a 1−0 binary as shown in Table 2. It should be noted that because we did not test for recovery of the worms in drug-free media after each day, we cannot be certain that the worms were truly dead or simply paralyzed. Following the score transformation, for each well in the experiment for each day the fraction survival of the well was found by weighting the number of worms of each score by its assigned score and summing those weights then dividing by the total number of worms in the well (e.g.,: [(0 x number of worms scored 0) + (1 x number of worms scored 1)] / total number of worms in the well). Note that all wells were assigned a fraction survival of 1 at day 0 (when worms were added to the well plate, the day before observation/scoring began). The fraction survivals from related concentration wells were averaged across all relevant same-life-stage/drug-treatment experiments. If an experiment was performed n = 5 times, the average was taken across 15 wells (3 replicates per experiment x 5 experimental runs). The resulting averages were then graphed as line graphs, with fraction survival on the y-axis, days on the x-axis, and line color denoting the drug concentration.

### IT_50_ and LT_50_

The IT_50_ is the time to 50% inhibition, and the LT_50_ is the time to 50% lethality. The IT_50_ was found for 3-2-1-0 and 2-1-0 scoring, while the LT_50_ was found for 1−0 scoring. Specifically, the IT_50_ is finding the day at which at least 50 percent of worms were scored at a 2, 1, or 0 in the 3-2-1-0 scoring system (as anything that is not a 3 is inhibited) and the day at which at least 50 percent of worms were scored at a 1 or 0 in the 2-1-0 scoring system (as anything that is not a 2 is inhibited); the LT_50_ is finding the day at which at least 50 percent of worms were scored as a 0 in the 1−0 scoring system. IT_50_ and LT_50_ analyses fall into the class of survival analyses and produce Kaplan-Meier curves [48,49] where inhibition or mortality is the “event” for which the worms are “at risk”.

For each IT_50_ and LT_50_ analysis, a representative experiment was first selected as the one that most closely resembled the average fraction alive and motility score graph, as determined by visual inspection by a subset of the authors. For each drug concentration used to treat the worms, the triplicate wells were pooled, and the analysis was used to determine the number of worms at risk of being inhibited or dead. For the IT_50_ analysis, these were worms that had been scored as 3’s in the 3-2-1-0 scoring system or scored as 2’s in the 2-1-0 scoring system; for the LT_50_ analysis, these were worms that had been scored as 1’s in the 1−0 scoring system. As previously described, we completed the analysis in GraphPad Prism [27] and report the data here. As is standard practice in survival analyses, once a worm experiences the event (inhibition or mortality), that event is considered irreversible for analytical purposes. Thus, for example, if a worm was scored a 2 on one day, but appeared healthy and scored a 3 the next day, within this analysis, the recovery is not noted, as the worms at risk decrease monotonically over time (i.e., stays the same or is lower than the day before but never increases). Finally, the number at risk was transformed into a percentage of the total worms in the pooled wells for that concentration. The percentage of worms at risk (uninhibited or alive) was plotted (on the y-axis) for each day across the length of the experiment (with day on the x-axis), and we found the day at which the line crossed 50%. If the line did not cross 50%, the IT_50_/LT_50_ was undefined and was noted with a “U”. This analysis was conducted independently for each concentration in the representative experiment for each drug/life stage assayed.

### IC_50_ and LC_50_

The IC_50_ is the concentration at which 50% inhibition of worm growth is observed, and the LC_50_ is the concentration at which 50% lethality is observed, both on the 4^th^ day of the experiment. We chose the 4^th^ day because it is the midpoint of the 7-day experiment. Specifically, the IC_50_ is finding the concentration at which at least 50 percent of worms are scored at a 2, 1, or 0 in the 3-2-1-0 scoring system (as anything that is not a 3 is inhibited) and the concentration at which at least 50 percent of worms are scored at a 1 or 0 in the 2-1-0 scoring system (as anything that is not a 2 is inhibited); the LC_50_ is finding the concentration at which 50 percent of worms are scored as a 0 in the 1−0 scoring system. A curve-fitting, non-linear regression approach is used with the IC_50_ and LC_50_ analyses.

As done previously, we completed the analysis for these in GraphPad Prism (https://www.graphpad.com/guides/prism/latest/curve-fitting/reg_asymmetric_dose_response_ec.htm) using a four-parameter logistic (4PL) nonlinear regression model [27], and report the data here. The value that we are interested in and report is the concentration at which the response is 50% for these plots. If the line does not cross 50%, we report that the IC_50_ exceeds the highest assayed concentration. If the line crosses 50% but somewhere between the controls and the lowest assayed concentration, we report that the IC_50_ is less than the lowest assayed concentration.

### Statistical analysis

The analysis was performed in GraphPad Prism. A two-way ANOVA with Dunnett test (p < 0.05) was performed for the motility index score graphs and the fraction alive graphs at every time point, for every concentration, with a confidence interval of 95%. All p-values are in S2 Table.

## Results and discussion

We present drug-by-drug results below. We then provide a comparative synthesis that ranks anthelmintic efficacy, examines stage-dependent susceptibility patterns, and discusses translational implications for tissue helminth infections and environmental control strategies.

### Effects of pyrantel on L1 *C. elegans*

**Fig 1:**
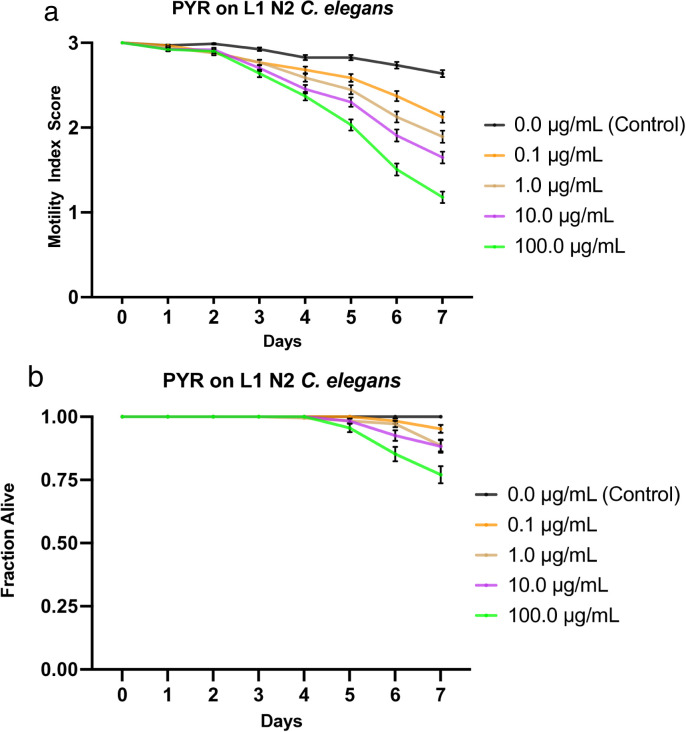
Effects of pyrantel (PYR) on L1 N2 *C. elegans.* a) Graph of average sample health rating utilizing motility index scale (0-3). b) Graph of average sample fraction alive. “3” represents a parasite with vigorous movement similar to control no drug; “2” represents a parasite with whole-body movements (observed without external stimulus) significantly slower than control no drug; “1” represents a parasite that was not moving on its own but moved when touched with a probe (tested at three different body locations); and 0 represents a worm that did not move even when prodded. Each score (motility and fraction alive) for each concentration was compared with the control on the same day, every day, and analyzed using two-way ANOVA with Dunnett’s test. Too many comparisons were statistically significant to denote with asterisks, so we report that information in Supplementary S2 Table. 100.0 μg/mL = 0.02 mM.

We treated L1 *C. elegans* with increasing doses of pyrantel (0.1, 1.0, 10, and 100 µg/mL). The worms were monitored at roughly the same time daily for 7 days, during which their motility was scored and compiled into a graphical visualization (Fig 1a). Mortality was also recorded daily (health rating scores of 0) and used to analyze the fraction of worms still alive, which was graphically represented (Fig 1b). These data show a clear negative correlation between drug concentration and worm health, as analyzed through the motility index score. Worms exposed to four concentrations caused a steady, stratified decline in motility based on drug dosage, while the worms under control conditions remained at over 2.5 motility score throughout the experiment (Fig 1a). By day 7, in order of increasing concentration starting with the control, the motility scores averaged 2.64, 2.12, 1.89, 1.65, and 1.18 (Fig 1a). Significant deviation from the control was not noted until day 4, while considerable separation in efficacy among the concentrations was evident by day 5. No mortality was observed among any of the worms in any condition until day 5, when the highest concentration sample fell to just above 95% survival (Fig 1b). By day 7, the 100 µg/mL concentration wells averaged approximately 77% survival, while the following two highest concentrations, 10 and 1 µg/mL, respectively, had approximately 88% survival. At the lowest concentration (0.1 µg/mL), survival dropped to ~95% on the 7^th^ day of drug exposure, while the worms under control conditions remained at 100% survival throughout the week (Fig 1b). The IT_50_ and LT_50_ data for each drug and concentration, including pyrantel, were compiled and presented in Table 3.

**Table 3:** IT_50_ and LT_50_ values for *C. elegans* on different anthelmintics (days).

Pyrantel (PYR) - L1											
0 µg/mL		0.1 µg/mL		1.0 µg/mL		10 µg/mL		100 µg/mL		1000 µg/mL	
LT_50_	IT_50_	LT_50_	IT_50_	LT_50_	IT_50_	LT_50_	IT_50_	LT_50_	IT_50_	LT_50_	IT_50_
U	U	U	U	U	7	U	7	U	6	N/A	N/A
Nitazoxanide (NTZ) - L1											
0 µg/mL		0.1 µg/mL		1.0 µg/mL		10 µg/mL		100 µg/mL		1000 µg/mL	
LT_50_	IT_50_	LT_50_	IT_50_	LT_50_	IT_50_	LT_50_	IT_50_	LT_50_	IT_50_	LT_50_	IT_50_
U	U	U	6	U	5	U	4	7	4	N/A	N/A
Ivermectin (IVM) - L1											
0 µg/mL		0.1 µg/mL		1.0 µg/mL		10 µg/mL		100 µg/mL		1000 µg/mL	
LT_50_	IT_50_	LT_50_	IT_50_	LT_50_	IT_50_	LT_50_	IT_50_	LT_50_	IT_50_	LT_50_	IT_50_
U	6	U	1	U	1	U	1	5	1	N/A	N/A
Albendazole (ALB) - L1											
0 µg/mL		0.1 µg/mL		1.0 µg/mL		10 µg/mL		100 µg/mL		1000 µg/mL	
LT_50_	IT_50_	LT_50_	IT_50_	LT_50_	IT_50_	LT_50_	IT_50_	LT_50_	IT_50_	LT_50_	IT_50_
U	U	U	6	U	4	U	4	U	2	N/A	N/A
Mebendazole (MBZ) - L1											
0 µg/mL		0.1 µg/mL		1.0 µg/mL		10 µg/mL		100 µg/mL		1000 µg/mL	
LT_50_	IT_50_	LT_50_	IT_50_	LT_50_	IT_50_	LT_50_	IT_50_	LT_50_	IT_50_	LT_50_	IT_50_
U	U	U	7	U	6	U	6	U	4	N/A	N/A
Mebendazole (MBZ) - L4											
0 µg/mL		0.1 µg/mL		1.0 µg/mL		10 µg/mL		100 µg/mL		1000 µg/mL	
LT_50_	IT_50_	LT_50_	IT_50_	LT_50_	IT_50_	LT_50_	IT_50_	LT_50_	IT_50_	LT_50_	IT_50_
U	U	U	7	U	5	U	5	U	4	U	2

IT_50_ and LT_50_ values (in days) for *C. elegans* on different anthelmintics are reported here for the L1 stage for pyrantel (100.0 μg/mL = 0.2 mM), nitazoxanide (100.0 μg/mL = 0.3 mM), ivermectin (100.0 μg/mL = 0.1 mM), albendazole (100.0 μg/mL = 0.4 mM), and mebendazole (100.0 μg/mL = 0.3 mM), and the L4 stage for mebendazole (1000.0 μg/mL = 3.4 mM) only. They are color-coded to match the line graphs (Figures a and b 1-6) for the same concentration. U = undefined (if the line did not cross 50%, the IT_50_/LT_50_ was undefined, which is notated with a “U”). N/A = not applicable because it was not performed.

**Fig 2:**
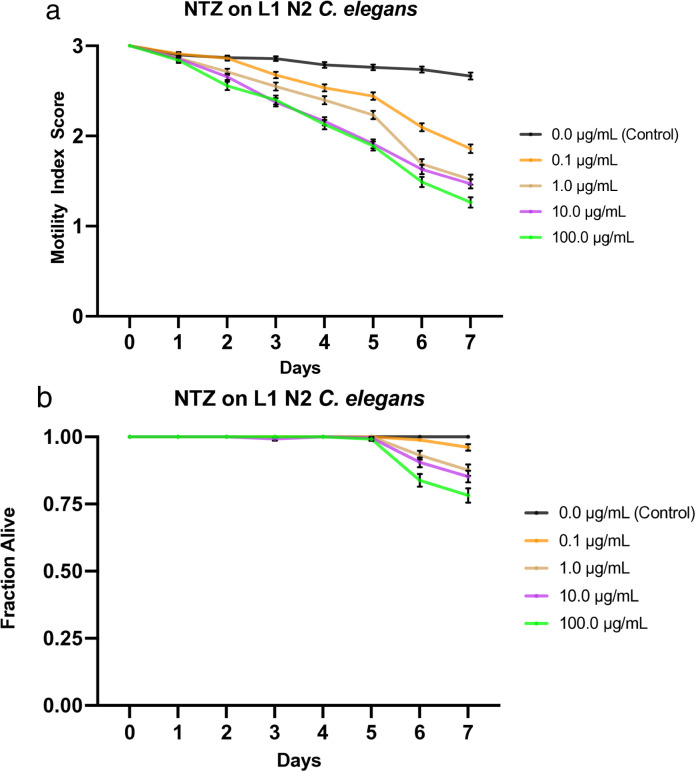
Effects of nitazoxanide (NTZ) on L1 N2 *C. elegans.* a) Graph of average sample health rating utilizing motility index scale (0-3). b) Graph of average sample fraction alive. “3” represents a parasite with vigorous movement similar to control no drug; “2” represents a parasite with whole-body movements (observed without external stimulus) significantly slower than control no drug; “1” represents a parasite that was not moving on its own but moved when touched with a probe (tested at three different body locations); and 0 represents a worm that did not move even when prodded. Each score (motility and fraction alive) for each concentration was compared with the control on the same day, every day, and analyzed using two-way ANOVA with Dunnett’s test. Too many comparisons were statistically significant to denote with asterisks, so we report that information in Supplementary S2 Table. 100.0 μg/mL = 0.3 mM.

**Fig 3:**
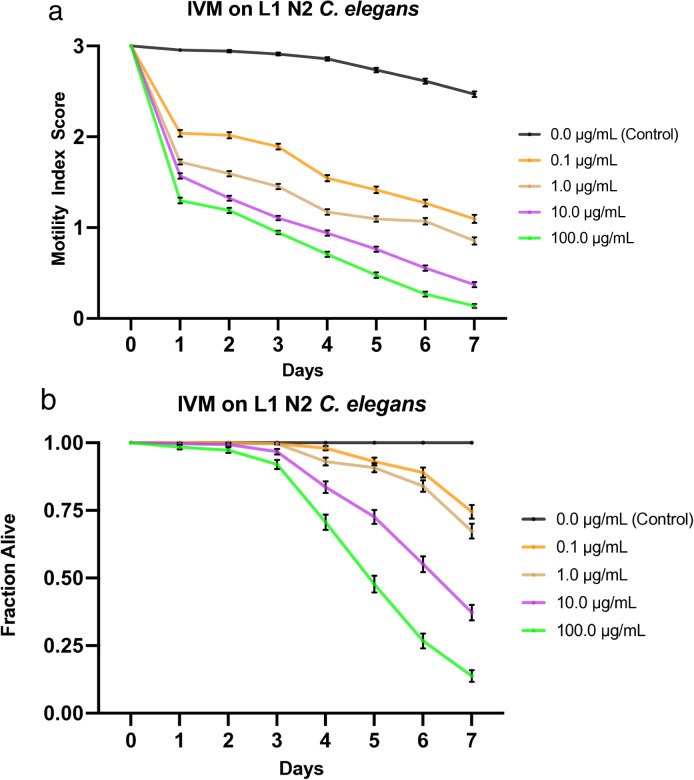
Effects of ivermectin (IVM) on L1 N2 *C. elegans.* a) Graph of average sample health rating utilizing motility index scale (0-3). b) Graph of average sample fraction alive. “3” represents a parasite with vigorous movement similar to control no drug; “2” represents a parasite with whole-body movements (observed without external stimulus) significantly slower than control no drug; “1” represents a parasite that was not moving on its own but moved when touched with a probe (tested at three different body locations); and 0 represents a worm that did not move even when prodded. Each score (motility and fraction alive) for each concentration was compared with the control on the same day, every day, and analyzed using two-way ANOVA with Dunnett’s test. Too many comparisons were statistically significant to denote with asterisks, so we report that information in Supplementary S2 Table. 100.0 μg/mL = 0.1 mM.

**Fig 4:**
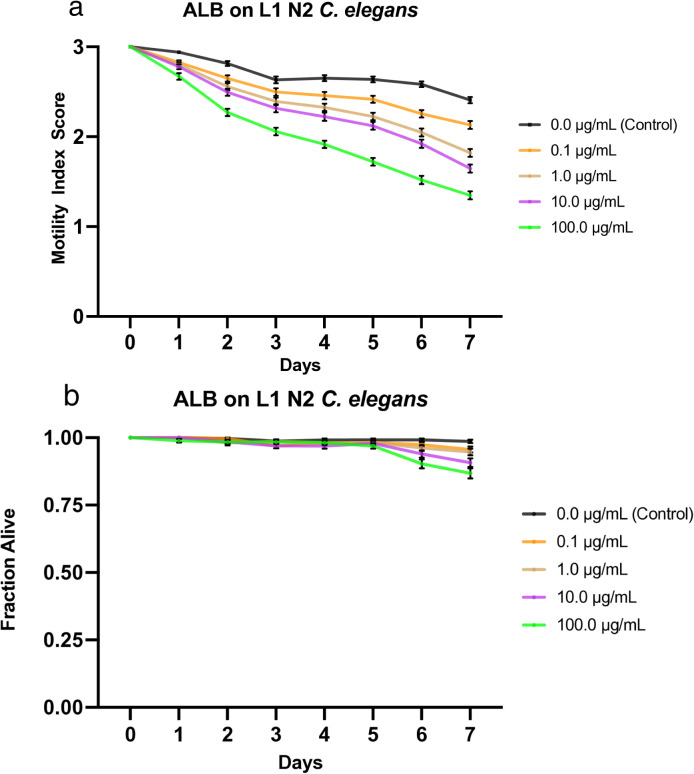
Effects of albendazole (ALB) on L1 N2 *C. elegans.* a) Graph of average sample health rating utilizing motility index scale (0-3). b) Graph of average sample fraction alive. “3” represents a parasite with vigorous movement similar to control no drug; “2” represents a parasite with whole-body movements (observed without external stimulus) significantly slower than control no drug; “1” represents a parasite that was not moving on its own but moved when touched with a probe (tested at three different body locations); and 0 represents a worm that did not move even when prodded. Each score (motility and fraction alive) for each concentration was compared with the control on the same day, every day, and analyzed using two-way ANOVA with Dunnett’s test. Too many comparisons were statistically significant to denote with asterisks, so we report that information in Supplementary S2 Table. 100.0 μg/mL = 0.4 mM.

**Fig 5:**
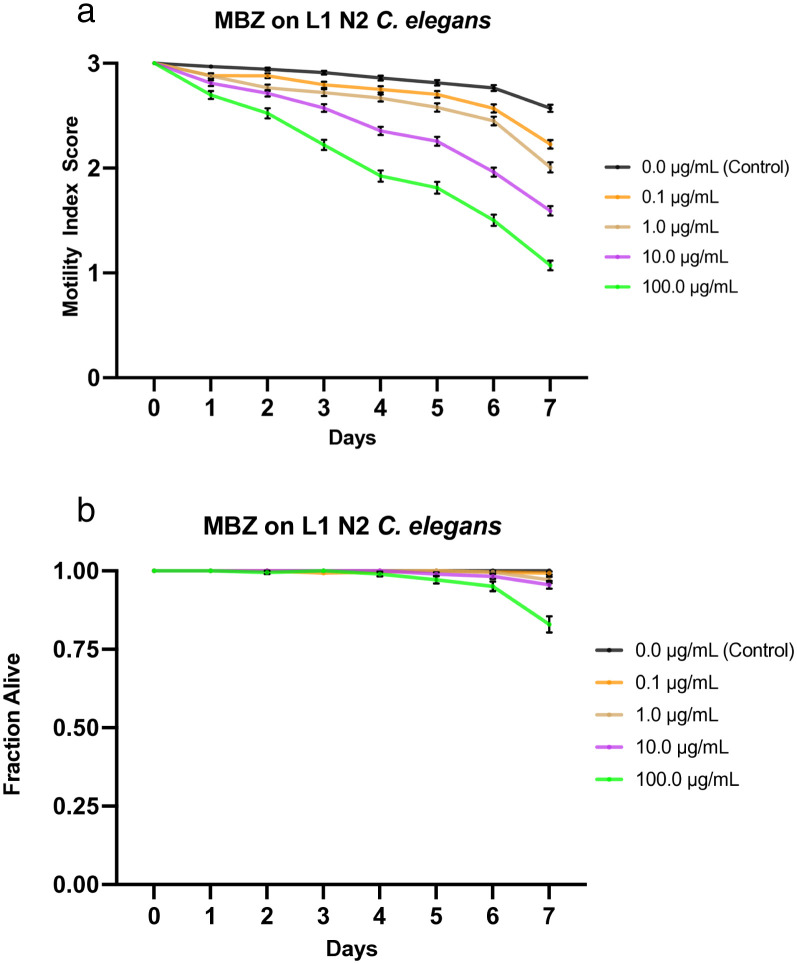
Effects of mebendazole (MBZ) on L1 N2 *C. elegans.* a) Graph of average sample health rating utilizing motility index scale (0-3). b) Graph of average sample fraction alive. “3” represents a parasite with vigorous movement similar to control no drug; “2” represents a parasite with whole-body movements (observed without external stimulus) significantly slower than control no drug; “1” represents a parasite that was not moving on its own but moved when touched with a probe (tested at three different body locations); and 0 represents a worm that did not move even when prodded. Each score (motility and fraction alive) for each concentration was compared with the control on the same day, every day, and analyzed using two-way ANOVA with Dunnett’s test. Too many comparisons were statistically significant to denote with asterisks, so we report that information in Supplementary S2 Table. 100.0 μg/mL = 0.3 mM.

**Fig 6:**
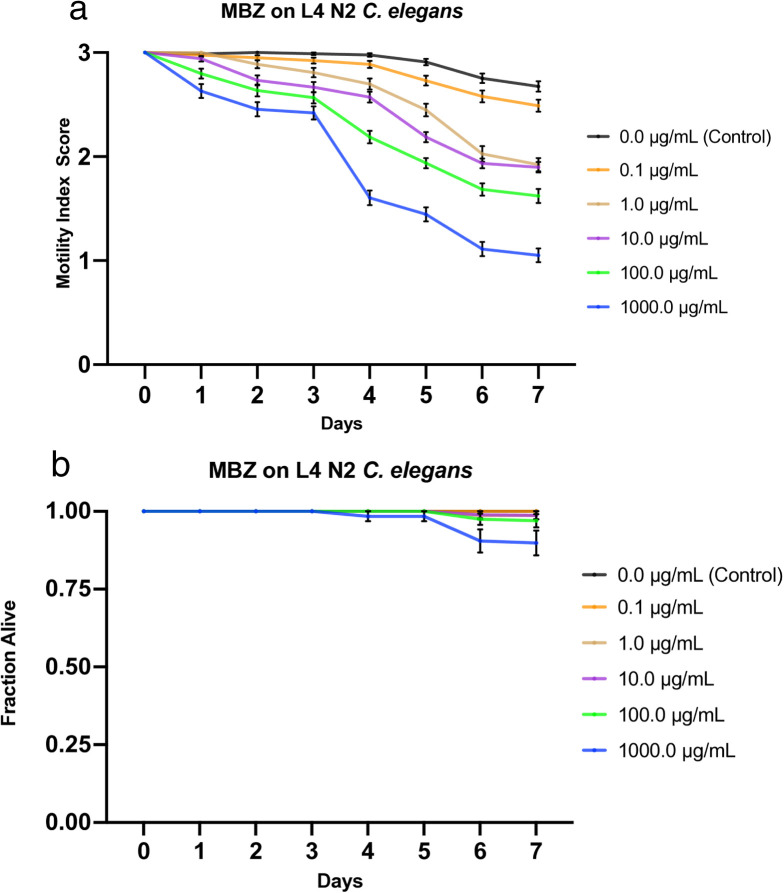
Effects of mebendazole (MBZ) on L4 N2 *C. elegans.* a) Graph of average sample health rating utilizing motility index scale (0-3). b) Graph of average sample fraction alive. “3” represents a parasite with vigorous movement similar to control no drug; “2” represents a parasite with whole-body movements (observed without external stimulus) significantly slower than control no drug; “1” represents a parasite that was not moving on its own but moved when touched with a probe (tested at three different body locations); and 0 represents a worm that did not move even when prodded. Each score (motility and fraction alive) for each concentration was compared with the control on the same day, every day, and analyzed using two-way ANOVA with Dunnett’s test. Too many comparisons were statistically significant to denote with asterisks, so we report that information in Supplementary S2 Table. 1000.0 μg/mL = 3.4 mM.

LT_50_ values for all concentrations were undefined. The IT_50_ was undefined for the control, defined as day 7 for both 1.0 and 10 µg/mL, and day 6 for 100 µg/mL (Table 3). The values reported in Table 3 are derived from a single representative experiment and should be interpreted alongside the averaged motility and fraction-alive data (Figs 1–6) for a complete understanding of drug efficacy. The IC_50_ and LC_50_ data for each drug, including pyrantel, were compiled and presented in Table 4.

**Table 4:** IC_50_ and LC_50_ values for different anthelmintics (μg/mL) on *C. elegans* at day 4.

Drug	LC_50_	IC_50_ (3−0 scoring)	IC_50_ (2−0 scoring)	IC_50_ Weaver *et al*. (L4)
Pyrantel	>100.0	>100.0	>100.0	0.375
Nitazoxanide	>100.0	0.640	>100.0	<0.1
Ivermectin	>100.0	<0.1	<0.1	<0.1
Albendazole	>100.0	0.989	>100.0	1.4
Mebendazole (L1)	>100.0	4.265	>100.0	N/A
Mebendazole (L4)	>1000.0	13.335	>1000.0	N/A

IC_50_ and LC_50_ values (μg/mL) at day 4 on L1 stage *C. elegans.* Using the anthelmintics pyrantel, nitazoxanide, ivermectin, albendazole, and mebendazole (performed on both L1 and L4 stage *C. elegans*), we report the IC_50_ and LC_50_ values from the middle day of the experiments. N/A = not applicable because it was not performed.

The LC_50_ was determined to be greater than 100 µg/mL, the highest concentration tested. Additionally, the IC_50_ value was undefined at day 4 of the experiment, indicating that a concentration greater than 100 µg/mL is required (Table 4). See S7 Fig for a graphical representation of these values.

Compared with the L4 data in Weaver *et al*. (2017) [27], the L1 worms appear significantly less sensitive to the drug. This is evident when comparing the average motility index scores: the highest drug concentration tested in our study (100 µg/mL) exhibited motility effects comparable to those of the lowest drug concentrations (0.1 and 1 µg/mL) tested on the L4s [27]. While the L1 worms studied here required 5 days of exposure to 100 µg/mL of drug before averaging approximately a 2 for motility, the L4 worms had dipped below a 2 on the first day of scoring when exposed to the same drug concentration, further emphasizing the increased susceptibility of the L4s to the drug. Comparing effects on mortality also shows similar differences between the two larval stages, with L4 worms appearing much more sensitive to the drug. For instance, L1s exposed to 100 µg/mL of drug averaged ~77% survival by the final day, while the L4 worms exposed to the same drug concentration averaged only 25% survival by day 7. Mortality is also observed earlier among the L4s, with all tested drug concentrations averaging 85%−95% survival by day 3 (even approaching 5% mortality by day 1 at 100 µg/mL). In contrast, mortality was not observed among the L1s until day 5, and to a much lesser extent [27]. Furthermore, while the L1 worms provided undefined values, the L4 worms exposed to 10 and 100 µg/mL of drug yielded LT_50_ values of days 7 and 6, respectively. Similarly, the IT_50_ values for the L4 worms were significantly earlier than those for the L1 worms—up to 5 days earlier at the highest concentration (100 µg/mL), indicating earlier inhibition. Additionally, the IC_50_ value on day 4 for the L1 worms was defined as >100 µg/mL as opposed to 0.375 µg/mL yielded by the L4 worms [27] (Table 4) (S7 Fig).

### Effects of nitazoxanide on L1 *C. elegans*

We treated L1 *C. elegans* with increasing doses of nitazoxanide (0.1, 1.0, 10, and 100 µg/mL) and monitored the worms at roughly the same time each day for 7 days, during which their motility was recorded and compiled into a graphical visualization (Fig 2a). Mortality was also reported (health rating scores of 0) and used to analyze the fraction of worms still alive each day, which was graphically represented (Fig 2b). Intoxication with nitazoxanide showed a dose-dependent effect on worm motility, as evidenced by a negative correlation between drug dose and motility. The worms under experimental conditions exhibited a mostly stratified decline in motility. However, there was overlap between the 10 and 100 µg/mL concentrations throughout the week, and between the 1.0 µg/mL concentration on the final 2 days. All experimental conditions averaged below 2 for motility by day 7, while the worms under control conditions averaged just under 3 by day 7. In order of increasing concentration, starting with the control, the motility scores averaged 2.67, 1.86, 1.52, 1.47, and 1.26 by the 7^th^ day (Fig 2a). Significant deviation from the control was apparent on day 2 (day 3 for the 0.1 µg/mL concentration). In comparison, considerable separation in efficacy among the experimental concentrations was evident by day 3 (apart from the 10 and 100 µg/mL concentrations, which continued to overlap until day 6). Furthermore, worms under all four experimental conditions exhibited mortality by day 6, with survival reduced to 84% and 78% at the highest drug concentration after 6 and 7 days of drug exposure, respectively, while worms under control conditions remained at 100% survival throughout the week. LT_50_ values for all concentrations were undefined, except for the 100 µg/mL concentration, which was defined as day 7. The IT_50_ was undefined for the control, defined as day 6 for 0.1 µg/mL, day 5 for 1.0 µg/mL, and day 4 for both 10 and 100 µg/mL (Table 3). The LC_50_ value on day 4 was determined to be greater than 100 µg/mL, the highest concentration tested. Additionally, the IC_50_ value on day 4 was between 0.1 µg/mL and 1.0 µg/mL, with the value likely approximately 0.640 µg/mL, determined by nonlinear best-fit analysis (Table 4). See S8 Fig for a graphical representation of these values.

Compared with the L4 data in Weaver *et al*. (2017) [27], the L1 worms appear slightly less sensitive to the drug. This is made evident when comparing the average motility index scores—the highest drug concentration tested on the L1s (100 µg/mL) exhibited comparable motility effects to the lowest drug concentration tested on the L4s (0.1 µg/mL), ending the week just under 1.3 motility, while the L4s exposed to 100 µg/mL averaged ~1 by day 7. Similarly, the two larval stages differ little in their effects on mortality. The L4s exhibit a slightly earlier onset of mortality than the L1s (days 4 and 6, respectively); however, the fractions are comparable by the 7^th^ day, with the L1s showing a slightly more pronounced stratification by drug concentration. Both larval stages provided undefined LT_50_ values for all tested concentrations, except for the 100 µg/mL concentration tested on the L1s, which yielded a value of 7; however, this is based on a representative experiment and should be analyzed in congruence with the other methods of data analysis to gain a complete understanding. The IT_50_ values for all four drug concentrations tested on the L4 worms (0.1, 1, 10, and 100 µg/mL) were defined as day 3, sooner than any IT_50_ value specified for the L1s— even the highest concentration (100 µg/mL), which was defined as day 4. Additionally, the IC_50_ value on day 4 for the L1 worms was determined as 0.640 µg/mL as opposed to <0.1 µg/mL yielded by the L4 worms [27] (Table 4) (S8 Fig).

### Effects of ivermectin on L1 *C. elegans*

We treated L1 *C. elegans* with increasing doses of ivermectin (0.1, 1.0, 10, and 100 µg/mL) and monitored the worms at roughly the same time each day for 7 days, during which their motility was scored and compiled into a graphical visualization (Fig 3a). Furthermore, mortality was noted (health rating scores of 0) and used to analyze the fraction of worms still alive daily, which was also graphed (Fig 3b). Worms exposed to ivermectin exhibit a strong, dose-dependent effect, as evidenced by the negative correlation between drug dose and worm motility. Similarly, there is a negative correlation between drug dose and worm survival. Unlike the other drugs tested in this study, all drugged worms had a motility score of ≤2 on the first day. At the same time, the worms under control conditions averaged a motility score of just below 3. As the experiments continued, motility declined steadily, stratified by drug concentration, and by day 7, all non-control worms were below a motility score of 1.1 (Fig 3a). Significant deviance from the control and noticeable separation in efficacy were apparent from day 1.

Furthermore, all four experimental concentrations exhibited significant mortality, while the control worms remained alive at 100%. The lowest concentration sample (0.1 µg/mL) averaged ~75% survival by day 7, 67% survival for the next highest concentration (1.0 µg/mL), 37% survival in the next highest (10 µg/mL), and down to 14% survival for the highest concentration sample (100 µg/mL) (Fig 3b). LT_50_ values for all concentrations were undefined, except for the 100 µg/mL concentration, which was defined as day 5. As with L4s [27], the motility scoring system was beneficial here to differentiate dead worms from very sick worms; without such a sensitive system, very sick worms would have surely been counted as dead. The IT_50_ was defined as day 6 for the control compared to day 1 for worms under all experimental conditions (Table 3). The LC_50_ was defined as greater than 100 µg/mL, the highest concentration tested. Additionally, the IC_50_ value on day 4 was < 0.1 µg/mL (Table 4). See S9 Fig for a graphical representation of these values.

Compared with the L4 data in Weaver *et al*. (2017) [27], the L1 worms appear slightly less sensitive to the drug. While the final motility scores at the higher doses do not differ much between the two larval stages, the trends become evident when analyzing the data. Both larval stages exhibited a severe drop in motility on the first scoring day; however, the drop was much more significant among the L4 worms. Except for the 0.1 µg/mL drug concentration, all other tested concentrations averaged ~1 motility score on day 1 for the L4 worms. In contrast, the L1 worms exposed to any drug concentration averaged 1.3-2 on the first day of scoring. Following the first scoring day, the L4 worms exhibited a gradual decline in motility for the remainder of the week, while the L1 worms maintained a steeper, clearly stratified decline. Further, the final motility scores for L4 at the lower concentrations were lower. Similarly, when comparing effects on mortality, the two larval stages share many features, exhibiting comparable fraction-alive values and general trends. While the only LT_50_ value for the L1 worms was observed at the highest drug concentration (100 µg/mL), the L4 worms exhibited a defined value at each drug concentration (1, 10, and 100 µg/mL), except at the lowest (0.1 µg/mL). The IT_50_ values were identical (day 1) across all drug concentrations for both larval stages, except for the lowest concentration (0.1 µg/mL) tested on L4 worms, which was defined as day 2 (Table 3). Additionally, the IC_50_ value on day 4 for both larval stages was less than 0.1 µg/mL [27] (Table 4) (S9 Fig).

### Effects of albendazole on L1 *C. elegans*

We treated L1 *C. elegans* with increasing doses of albendazole (0.1, 1.0, 10, and 100 µg/mL) and monitored the worms at roughly the same time each day for 7 days, during which their motility was scored and compiled into a graphical visualization (Fig 4a). Mortality was also noted (health rating scores of 0) and used to analyze the fraction of worms still alive daily, which was graphically represented (Fig 4b). These data show a clear negative correlation between drug concentration and worm health, as analyzed through the motility index score. Worms exposed to four concentrations exhibited a steady, stratified decline in motility while worms under control conditions remained at over 2.4 motility score throughout the experiment (Fig 4a). All drugged worms averaged under a motility score of 2 by day 7, except for the lowest concentration sample (0.1 µg/mL), which averaged just above 2. Like the Weaver results, although the drugged worms became unhealthier throughout the week, albendazole did not kill many *C. elegans*, as even the sample dosed with the highest concentration (100 µg/mL) had 87% still alive by day 7, followed by 91% (10 µg/mL), and 95% survival for the lower concentrations (0.1 and 1.0 µg/mL) (Fig 4b). Furthermore, LT_50_ values for all concentrations were undefined. The IT_50_ was undefined for the control, defined as day 6 for 0.1 µg/mL, day 4 for both 1.0 and 10 µg/mL, and day 2 for 100 µg/mL (Table 3). The LC_50_ was determined to be greater than 100 µg/mL, the highest concentration tested. Additionally, the IC_50_ value on day 4 was determined to be very slightly less than 1.0 µg/mL (Table 4). See S10 Fig for a graphical representation of these values.

Compared with the L4 data in Weaver *et al*. (2017) [27], the L1 worms showed a similar susceptibility to the drug. While the final motility scores do not differ much between the two larval stages, minor differences are observed in the trend analysis. The L1 worms exhibited more pronounced stratification by concentration and a steadier decline in motility, as opposed to slightly greater fluctuation and a gradual decline in the latter half of the week observed in the L4 data. While each drug concentration produced distinct results in the L1 worms, the L4 worms showed nearly identical motility responses to the highest drug concentrations (10 and 100 µg/mL) throughout most of the 7 days. Similarly, when comparing effects on mortality, the two larval stages share many features, exhibiting comparable fraction-alive values and general trends. The L4s have slightly lower fractions alive by the 7^th^ day and a slightly earlier onset of mortality compared to the L1s. The LT_50_ values for both larval stages were undefined. The IT_50_ values were quite similar, with the only differences being present at the lowest and highest concentrations (0.1 and 100 µg/mL, respectively), during which the L4’s values were one day earlier and later, respectively (Table 3). Additionally, the IC_50_ values on day 4 for both larval stages were ~1.0 µg/mL, with the L1 value being defined slightly lower at .989 µg/mL compared to 1.4 µg/mL for the L4 worms [27] (S10 Fig) (Table 4).

### Effects of mebendazole on L1 *C. elegans*

We treated L1 *C. elegans* with increasing doses of mebendazole (0.1, 1.0, 10, and 100 µg/mL) and monitored the worms at roughly the same time each day for 7 days, during which their motility was scored and compiled into a graphical visualization (Fig 5a). Mortality was also noted (health rating scores of 0) and used to analyze the fraction of worms still alive daily, which was graphically represented (Fig 5b). These data show a clear negative correlation between drug concentration and worm health, as analyzed through the motility index score. Worms exposed to four concentrations showed a steady, stratified decline in motility based on drug dosage, while the control worms remained above 2.5 motility score throughout the experiment (Fig 5a). In order of increasing concentration, starting with the control, the motility scores averaged 2.57, 2.23, 2.01, 1.59, and 1.07 by the 7^th^ day (Fig 5a). Like with albendazole (Fig 4b), mebendazole does not kill many worms, as even the sample dosed with the highest concentration (100 µg/mL) had just over 82% still alive on day 7, while all other concentrations exhibited little to no mortality, remaining above 95% survival by day 7 (Fig 5b). Furthermore, LT_50_ values for all concentrations were undefined. The IT_50_ was undefined for the control, defined as day 7 for 0.1 µg/mL, day 6 for both 1.0 and 10 µg/mL, and day 4 for 100 µg/mL (Table 3). The LC_50_ was determined to be greater than 100 µg/mL, the highest concentration tested. Additionally, the IC_50_ value on day 4 was determined to be ~ 4.265 µg/mL through nonlinear best-fit analysis (Table 4). See S11 Fig for a graphical representation of these values.

### Effects of mebendazole on L4 *C. elegans*

We treated L4 *C. elegans* with increasing doses of mebendazole (0.1, 1.0, 10, 100, and 1000 µg/mL) and monitored the worms at roughly the same time each day for 7 days, during which their motility was scored and compiled into a graphical visualization (Fig 6a). Mortality was also scored (health rating scores of 0) and used to analyze the fraction of worms still alive daily, which was also graphed (Fig 6b). L4 worms exposed to mebendazole exhibit a strong dose-dependent effect, as observed in the negative correlation between drug dose and worm motility. The *C. elegans* exhibited a stratified decline in motility with little overlap between the worms under different conditions. Worms under all experimental conditions, except for the lowest dose of 0.1 µg/mL, dipped below 2 for motility, with the highest concentration (1000 µg/mL) averaging nearly a 1 by the 7^th^ day, while worms under control conditions averaged just under a 3 by day 7. In order of increasing concentration, starting with the control, the motility scores averaged 2.67, 2.49, 1.92, 1.90, 1.62, and 1.05 by the 7^th^ day (Fig 6a). Furthermore, little to no mortality was observed among the entire population, as only the 1000 µg/mL concentration exhibited some mortality (10%) and only after the 6^th^ day, while the rest of the worms under the other conditions, as well as the control, remained at, or nearly at, 100% survival throughout the entirety of the week (Fig 6b). LT_50_ values for all concentrations were undefined. The IT_50_ was undefined for the control, defined as day 7 for 0.1 µg/mL, day 5 for both 1.0 µg/mL and 10 µg/mL, day 4 for 100 µg/mL, and day 2 for 1000 µg/mL (Table 3). The LC_50_ was defined as greater than 1000 µg/mL, the highest concentration tested. Additionally, the IC_50_ value on day 4 was slightly greater than 10 µg/mL, with the likely concentration estimated at 13.335 µg/mL by nonlinear best-fit analysis (Table 4). See S12 Fig for a graphical representation of these values.

We primarily see similarities when comparing the L1 to the L4 MBZ data, but a few differences stand out. The main difference is that L1 worms seem slightly more susceptible than L4 worms to most concentrations tested, except 1.0 µg/mL, with the greatest difference observed on day 7 at 100 µg/mL, where L1 worms had a motility score of 1.07 and L4 worms had a score of 1.62. Further, the motility graphs show how the data trends throughout the week. For all 4 concentrations in the L1 experiment, worm motility declined gradually until day 5, when the slopes steepened. This also seems to be generally true for the L4 experiments. The L4 experiment is the only one in which we used the 1000 µg/mL concentration (as in Weaver *et al*.), and the results were similar: it was stratified and showed the same trend as the lower concentrations, reaching a score of 1.05 on day 7. The motility index scoring followed a similar trend as the worms under the 100 µg/mL condition, but consistently had a lower score for each day; however, it had a much steeper slope between days 3 and 4. Regarding the fraction alive, the only noticeable difference was that on day 7, at 100 µg/mL, L1 had a lower fraction than L4 (.829 vs. .970, respectively), even lower than at 1000 µg/mL, which ended at .898.

Overall, in this study, we used 7-day liquid assays to evaluate the health and life of *C. elegans*. We did this across a range of anthelmintic concentrations. We then calculated and analyzed the IT_50_, LT_50,_ LC_50,_ and IC_50_ values for each anthelmintic to better understand efficacy over time. Across all drugs and concentrations tested, several key patterns emerged. Ivermectin had the greatest effect, followed by nitazoxanide and albendazole, with pyrantel and mebendazole showing comparatively lower efficacy. Stage-dependent susceptibility varied by drug, with L4 worms generally more susceptible than L1—most notably for pyrantel and nitazoxanide—contrary to our initial hypothesis.

Ivermectin showed the greatest effect among all experimental concentrations. At all tested drug concentrations, nearly the entire experimental population of worms was inhibited by day 1, highlighting the high efficacy of this drug on the health of the worms with an IC_50_ value on day 4 < 0.1 µg/mL (S9 Fig). Not only were they inhibited, but their size was also greatly diminished, similarly to the effect on L4s (not shown), and overall, the movement, as a 1 or 2, was strikingly less than that of other drugs. Nitazoxanide and albendazole exhibited the next-highest efficacy, with similar IT_50_ values and average motility graphs. The main difference between the two was evident at the highest and lowest concentrations: albendazole was more efficacious at higher concentrations, while nitazoxanide was more efficacious at lower concentrations. Additionally, albendazole exhibited a stratified, dose-dependent response, whereas nitazoxanide yielded more grouped results (Figs 2a, 4a). The observation that albendazole intoxicates *C. elegans* from the L1 stage onward further confirms the utility of *C. elegans* as a model for anthelmintic studies. Although they do not die, they are affected by the WHO MDA drug of choice, albendazole, becoming significantly less motile and thus obviously sick. The next-highest effect on worm motility was observed with pyrantel, followed very closely by mebendazole. Both pyrantel and mebendazole had similar IT_50_ and LT_50_ values (Table 3) and average motility graphs. While the worms exposed to mebendazole exhibited a more evident dose-dependent stratification of motility values and slightly higher efficacy in the higher concentration, the graphs for worms dosed with pyrantel exhibit a generally slightly steeper slope as well as increased mortality and thus somewhat more efficacious by slim margins (Figs 1a, 5a, 1b, 5b).

Regarding worm mortality, ivermectin had an apparent effect at all tested concentrations, with the lowest dose exhibiting an average survival of ~74%. In comparison, the worms in the highest concentration averaged ~14% survival by day 7 (Fig 3b). This starkly contrasts with the other drugs tested, during which the subsequent lowest average survival is approximately 77% by day 7 (Figs 1–5b). Additionally, ivermectin was one of the only drugs tested to provide a defined LT_50_ value, while nitazoxanide was the other. Although both drugs had a defined LT_50_ only at the highest concentration, it was day 5 for ivermectin and day 7 for nitazoxanide—further emphasizing the efficacy of ivermectin. This is likely due to the high affinity binding that ivermectin has for the glutamate gated chloride channels compared to other anthelmintics binding affinities for their respective targets [50], and potentially due to its pharmacokinetics and tissue penetration due to its lipophilic nature [51]. These results were expected, as the two drugs had the greatest impact on worm motility and health. Each of the remaining drugs had an undefined value for LT_50_ among all tested concentrations, thus implying that a higher dosage or more prolonged exposure to the drug is required for lethality. Additionally, the LC_50_ on day 4 was defined as greater than the highest drug concentration tested for each drug, suggesting that a higher concentration would be required to be sufficiently lethal by day 4.

Although the LT_50_ values may suggest that nitazoxanide is more lethal than pyrantel, these values are derived from a single representative experiment rather than averages of the collective data (as the motility and fraction alive graphs are). While these values provide snapshots that can quickly yield helpful information, their standalone use without accounting for the rest of the data may yield an incomplete understanding of the results. This is directly evident in the analysis of the fraction alive graphs (Figs 1b,2b), which show that pyrantel is slightly more effective at killing the worms, contrary to the LT_50_ values. As expected, nitazoxanide is followed by mebendazole, then by albendazole, in terms of lethality.

We hypothesized that L1 worms would be more susceptible than L4 worms. However, we observed the opposite: L4 worms were generally more susceptible, particularly to pyrantel and nitazoxanide. Mebendazole was the only exception, showing slightly greater efficacy against L1 worms at the highest concentration on day 7. These observed differences extended beyond the degree of health to the timing of effects across the experiments. This was especially evident with pyrantel, as there were notable differences in worm motility, fraction alive, and time to inhibition and mortality between the two larval stages, indicating that L4 worms were more susceptible. Nitazoxanide exhibited the next most substantial difference between the larval stages, following the same trend of L4s being slightly more susceptible to the drug than L1s, although to a much lesser extent than pyrantel. This was again evident in slightly lower final motility and fraction alive scores among the L4 worms, and in an earlier appearance of inhibition and mortality. While the final ivermectin results did not differ substantially between L1 and L4 larval stages, L4 worms exhibited lower scores earlier in the experiment than L1 worms. Similar comparisons were observed with albendazole intoxication—little difference in final scores; however, trends in the results, as well as various data analysis methods, show that the L4 worms exhibit a slightly earlier onset of inhibition that becomes more gradual as the week progresses, ending with scores comparable to those of the L1 worms.

Thus, while we predicted that L1 worms would be more susceptible, we did not find that in any of the anthelmintics tested (minus slightly with mebendazole on certain days/concentrations), and, strikingly, if there was a difference in susceptibility, it was the L4s that were more susceptible, again specifically most observed with pyrantel and nitazoxanide. The explanation for this is not totally clear although Bruckner *et al*., points out that, at least in rodents, susceptibility is difficult to predict based on age alone because toxicity seems to depend both on age as well as the compound itself due to differences in the metabolism, absorption, distribution, and elimination of the compound that coincide with a variation in anatomy, biochemistry, and physiology between age groups or life stages [52].

Specifically for *C. elegans*, we speculate that differences in efficacy could be due to differences across stages, including physiological differences, drug target expression, cuticle permeability, metabolic activity, drug uptake via feeding, or detoxification mechanisms. Physiological differences may make the drugs more efficacious in L4 larvae, as they undergo rapid growth and differentiation, particularly in reproductive organ development, which increases metabolic demand and thus drug susceptibility [53,54]. For drug target expression, the targets may be significantly more expressed in L4 than in L1 [55]. For example, L4 larvae, with more developed neuromuscular systems, likely express higher total levels of neuromuscular targets [56,57]. For cuticle permeability, changes in the cuticle across larval stages may affect anthelmintic uptake and, in turn, efficacy [58–62]. Indeed, both Hu *et al.* (2013) [63] and Ruiz-Lancheros *et al*. (2011) [64] suggest that the cuticle acts as a barrier to small molecules, a barrier that could be further magnified at different stages by physical differences in the cuticle. For example, Sundaram *et al*. describe the L1 stage as having longitudinal alae ridges, absent in the L4 stage. For metabolic activity, Burns *et al*., 2015 noted increased anthelmintic efficacy in L4 due to their increased energy demands with the discovery of a drug that targeted the electron transport chain [26], while Luz *et al*. (2017) noted high mitochondrial activity in L4, making them potentially more susceptible [65]. During feeding, L4 *C. elegans* exhibit vigorous pharyngeal pumping compared to L1, and drugs such as levamisole and ivermectin have been shown to significantly disrupt pharyngeal pumping, thereby affecting feeding [66]. Regarding detoxification mechanisms, Ardelli and Prichard (2013), Janssen *et al*. (2013), and David *et al*. (2016) showed that expression of P-glycoprotein (Pgp), a protein that detoxifies xenobiotics, decreases ivermectin efficacy. Thus, L1 may express higher levels of Pgp than L4 [67–69]. Each of these factors, individually or in combination, could have contributed to the increased efficacy of anthelmintics against L4 compared to L1. Similar research shows that genetic variation drives anthelmintic responses among wild strains of *C. elegans* [70,71].

Each of the outlined factors—severity of effect on motility, fraction alive, and timing in the appearance of those effects (is motility and fraction alive affected immediately following intoxication, or does it take a few days?) — all play a role in our overall understanding of the anthelmintics and help us understand ways in which to utilize them best. For instance, if we know that there is little to no difference in the effects observed when worms are exposed to 10 µg/mL versus 100 µg/mL of drug, then there would be little reason to administer doses higher than the 10 µg/mL since the effects of the drug plateau at a specific concentration and treating with a lower dose generally induces fewer side effects in the patient. Similarly, understanding the best drug for different larval stages will allow us to personalize treatments more effectively based on the presumed stage of infection, helping clear the infection more quickly and cost-effectively while combating observed resistance, even though most people are infected with multiple helminth stages.

Given the translational implications of our data, it is reasonable to begin future studies on various STH larval stages using anthelmintics with defined LT_50_ values at least at one concentration, in both *in vitro* and controlled environmental settings. For anthelmintics with undefined LT_50_ values across all concentrations, it is unclear what this will mean for their translational use, either for larval-stage infections or for environmental control. We reason that for larval-stage tissue infections, these anthelmintics will likely be sufficient for cure, as the host immune system will aid in their killing and removal, and because they all showed some reduction in health (highlighting the value of the health rating system). For potential environmental control, these anthelmintics will need to be tested on helminths *in vitro* and in controlled environmental settings. We predict that even without a host immune system to aid efficacy, these anthelmintics will reduce the infection rate. This is because even though the drugs did not kill these worms, they did cause them to become sick, as evidenced by slower movement (again demonstrating the value of the health rating system). This reduction in movement and overall health would almost certainly impair their ability to infect humans. Further, some of the anthelmintics that did not kill *C. elegans* do kill STHs *in vitro* [31], and this leads us to predict that the results seen here might be a conservative estimate of the efficacy that will be observed in larval stages of STHs *in vitro* and in the field.

Beyond the anthelmintics tested here, this systematic framework provides a comparative basis for prioritizing anthelmintic candidates and doses for validation in helminths. Given that most current anthelmintics primarily target adult helminths, our larval-stage susceptibility profiles may be particularly valuable for identifying therapeutics suited to tissue helminth infections, which typically occur at larval stages and for which treatment options remain suboptimal. The drug-to-drug and stage-to-stage comparisons we present enable researchers to make informed decisions about which compounds and concentrations warrant further investigation on a given parasitic species.

Importantly, our use of C. elegans as a model should be interpreted in light of cross-species validation studies. Hu *et al*. [31] demonstrated qualitatively and quantitatively similar drug effects in helminths but, critically, found albendazole to be less lethal to C. elegans than helminths. Similarly, Weaver *et al*. [27] observed dose-dependent health effects in C. elegans and confirmed that albendazole was not lethal. Together, these findings indicate that our C. elegans L1 susceptibility results likely represent a conservative lower bound on the expected efficacy against helminth larvae *in vitro* and potentially in the environment. This data-driven conservative interpretation strengthens the translational potential of our findings, while underscoring the necessity of species-specific validation in parasitic helminths under environmentally and clinically relevant conditions.

## Conclusions

Approximately 1.5 billion people worldwide suffer from STH infections. These infections disproportionately affect the poor and can cause people to become stuck in a so-called parasite trap, a vicious cycle of poverty and recurrent infection. The WHO currently recognizes two drugs for MDA: albendazole and mebendazole, having removed pyrantel and levamisole. Although these four drugs have been shown to be safe and effective in the past, an observed resistance has emerged due to only two unique mechanisms of action among the four drugs. This resistance calls for the research and further understanding of novel anthelmintics and new methods of helminth control to combat this endemic issue. Using *C. elegans* is a cost-effective and efficient way to screen for anthelmintics with reasonable confidence [26,27]. In this study, we compare the observed effects of current and former MDA-approved drugs with two mechanisms of action (β-tubulin inhibitors and nicotinic acetylcholine receptor agonists), as well as candidate MDA anthelmintics, to assess drug efficacy. While this study mainly focuses on L1 *C. elegans*, we also studied L4 worms to analyze variability in drug efficacy across larval stages and to fill a gap in information left by the Weaver *et al*. paper. Additionally, to our knowledge, we propose, for the first time, exploring the possibility of helping eradicate the endemic hookworm problem by treating contaminated soil. This novel control strategy should be used strategically, in addition to ongoing work to improve hygienic conditions and access to clean water, support drug discovery, enhance delivery methods/production [72], and utilize anthelmintics for people already infected with STHs.

Although we expected L1 worms to be more susceptible to anthelmintics than L4 worms, this was not the case in our results. We observe that there can be significant differences in how larval stages react to anthelmintics, and that these differences vary depending on the anthelmintic used. However, the worm stage does not matter significantly when comparing L1 to L4, as the data was relatively similar. We are unsure about the other stages but expect similar results, except for the egg stage, which could be quite different. Continuing to screen and test novel anthelmintics at different dosages can help us hone our efforts to find a safe, efficacious, and cost-effective solution to this neglected tropical disease (NTD). Testing these drugs across different larval stages will continue to expand our knowledge and help us identify the best target for each drug. For instance, finding a drug best suited to inhibit L1 worms, or even the eggs, may be a valuable preventive treatment or for tissue infections still in larval stages. In contrast, a drug targeting adult worms may be a better option for those already suffering from infection, as the adult stage is, by far, the longest stage during normal infection.

Although L4s were generally either equally or more susceptible to the anthelmintics than L1s, the L1s were susceptible to all drugs tested. The health-rating system from Hu *et al*. [31] and Weaver *et al*. [27] again proved valuable, as neither albendazole nor mebendazole killed the worms at any dose tested to a significant level, yet both caused a significant reduction in motility. We also note that ivermectin was much more efficacious than the other drugs tested.

Because hookworm is not infective until the L3 stage, we propose, to our knowledge, for the first time, using control methods such as spraying fields and water sources with anthelmintics to eliminate L1-L3 larval stages and prevent infection before it occurs. Our findings and strategy add further value to the strategy Partridge *et al*. (2018) suggested for spraying fields to control whipworm eggs [43]. Though this strategy could have some challenges, such as increased resistance and ecological repercussions, it would be a new and perhaps very powerful way to help fight the epidemic and improve the quality of life for countless people suffering from recurrent infections. Nunes *et al*. (2016), Goodenough *et al*. (2019), and de Souza and Guimares (2022) have documented potential ecological risks associated with anthelmintic exposure at environmentally realistic concentrations [73–75]. Further, the translation into a preinfective control will likely require different efficacy criteria, as the use of these drugs as therapeutics is aided by the host immune system in removing the parasites. Even so, using anthelmintics in a targeted way, for example, on “hot spots”, as Partridge suggests, could maximize the advantages of controlling helminth infection, while minimizing the ecological risks, and we recommend more extensive investigation and validation, especially in controlled field environments, should be pursued to support the feasibility of this strategy. In light of Hu *et al*. and Weaver *et al*., where benzimidazoles were less lethal to *C. elegans* than to parasitic nematodes [27,31], our *C. elegans* results here could be interpreted as conservative with respect to expected efficacy in hookworm and related STH larvae; nevertheless, controlled field assays for species‑specific infectivity, off‑drug recovery dynamics, and exposure‑stability remain required prior to any implementation. Expanding our arsenal of safe, effective anthelmintics for both environmental control and therapeutic treatment will help overcome resistance and end the cycle of infection that devastates a quarter of the global population.

## Supporting information

S1 Fig Effects of pyrantel (PYR) on L1 N2 *C. elegans.* Graph of average sample health rating utilizing motility index scale (0–2). “2” represents a parasite with whole-body movements (observed without external stimulus) significantly slower than control no drug; “1” represents a parasite that was not moving on its own but moved when touched with a probe (tested at three different body locations); and 0 represents a worm that did not move even when prodded.
(TIF)

S2 Fig Effects of nitazoxanide (NTZ) on L1 N2 *C. elegans.* Graph of average sample health rating utilizing motility index scale (0–2). “2” represents a parasite with whole-body movements (observed without external stimulus) significantly slower than control no drug; “1” represents a parasite that was not moving on its own but moved when touched with a probe (tested at three different body locations); and 0 represents a worm that did not move even when prodded.
(TIF)

S3 Fig Effects of ivermectin (IVM) on L1 N2 *C. elegans.* Graph of average sample health rating utilizing motility index scale (0–2). “2” represents a parasite with whole-body movements (observed without external stimulus) significantly slower than control no drug; “1” represents a parasite that was not moving on its own but moved when touched with a probe (tested at three different body locations); and 0 represents a worm that did not move even when prodded.
(TIF)

S4 Fig Effects of albendazole (ALB) on L1 N2 *C. elegans.* Graph of average sample health rating utilizing motility index scale (0–2). “2” represents a parasite with whole-body movements (observed without external stimulus) significantly slower than control no drug; “1” represents a parasite that was not moving on its own but moved when touched with a probe (tested at three different body locations); and 0 represents a worm that did not move even when prodded.
(TIF)

S5 Fig Effects of mebendazole (MEB) on L1 N2 *C. elegans.* Graph of average sample health rating utilizing motility index scale (0–2). “2” represents a parasite with whole-body movements (observed without external stimulus) significantly slower than control no drug; “1” represents a parasite that was not moving on its own but moved when touched with a probe (tested at three different body locations); and 0 represents a worm that did not move even when prodded.
(TIF)

S6 Fig Effects of mebendazole (MBZ) on L4 N2 *C. elegans.* Graph of average sample health rating utilizing motility index scale (0–2). “2” represents a parasite with whole-body movements (observed without external stimulus) significantly slower than control no drug; “1” represents a parasite that was not moving on its own but moved when touched with a probe (tested at three different body locations); and 0 represents a worm that did not move even when prodded.
(TIF)

S7 Fig Graphical visualization of data analysis methods for pyrantel (PYR) treated L1 N2. *C. elegans.* a-e) Graphs depicting LT_50_ (Purple), IT_50_ 3−0 scoring (Orange), and combined 3 & 2 (2−0 scoring) IT_50_ (Blue) values for worms exposed to increasing concentrations of the drug. Graphs correspond to values in Table 3 (3−0 scoring) and S1 Table (2−0 scoring). f-g) Graphs depicting IC_50_ values on day 4 utilizing 3−0 and 2−0 scoring, respectively. h) Graph depicting LC_50_ values on day 4. Graphs correspond to values in Table 4.
(TIF)

S8 Fig Graphical visualization of data analysis methods for nitazoxanide (NTZ) treated L1 N2. *C. elegans.* a-e) Graphs depicting LT_50_ (Purple), IT_50_ 3−0 scoring (Orange), and combined 3 & 2 (2−0 scoring) IT_50_ (Blue) values for worms exposed to increasing concentrations of the drug. Graphs correspond to values in Table 3 (3−0 scoring) and S1 Table (2−0 scoring). f-g) Graphs depicting IC_50_ values on day 4 utilizing 3−0 and 2−0 scoring, respectively. h) Graph depicting LC_50_ values on day 4. Graphs correspond to values in Table 4.
(TIF)

S9 Fig Graphical visualization of data analysis methods for ivermectin (IVM) treated L1 N2. *C. elegans.* a-e) Graphs depicting LT_50_ (Purple), IT_50_ 3−0 scoring (Orange), and combined 3 & 2 (2−0 scoring) IT_50_ (Blue) values for worms exposed to increasing concentrations of the drug. Graphs correspond to values in Table 3 (3−0 scoring) and S1 Table (2−0 scoring). f-g) Graphs depicting IC_50_ values on day 4 utilizing 3−0 and 2−0 scoring, respectively. h) Graph depicting LC_50_ values on day 4. Graphs correspond to values in Table 4.
(TIF)

S10 Fig Graphical visualization of data analysis methods for albendazole (ALB) treated L1 N2. *C. elegans.* a-e) Graphs depicting LT_50_ (Purple), IT_50_ 3−0 scoring (Orange), and combined 3 & 2 (2−0 scoring) IT_50_ (Blue) values for worms exposed to increasing concentrations of the drug. Graphs correspond to values in Table 3 (3−0 scoring) and S1 Table (2−0 scoring). f-g) Graphs depicting IC_50_ values on day 4 utilizing 3−0 and 2−0 scoring, respectively. h) Graph depicting LC_50_ values on day 4. Graphs correspond to values in Table 4.
(TIF)

S11 Fig Graphical visualization of data analysis methods for mebendazole (MBZ) treated L1 N2. *C. elegans.* a-e) Graphs depicting LT_50_ (Purple), IT_50_ 3−0 scoring (Orange), and combined 3 & 2 (2−0 scoring) IT_50_ (Blue) values for worms exposed to increasing concentrations of the drug. Graphs correspond to values in Table 3 (3−0 scoring) and S1 Table (2−0 scoring). f-g) Graphs depicting IC_50_ values on day 4 utilizing 3−0 and 2−0 scoring, respectively. h) Graph depicting LC_50_ values on day 4. Graphs correspond to values in Table 4.
(TIF)

S12 Fig Graphical visualization of data analysis methods for mebendazole (MBZ) treated L4 N2. *C. elegans.* a-f) Graphs depicting LT_50_ (Purple), IT_50_ 3−0 scoring (Orange), and combined 3 & 2 (2−0 scoring) IT_50_ (Blue) values for worms exposed to increasing concentrations of the drug. Graphs correspond to values in Table 3 (3−0 scoring) and S1 Table (2−0 scoring). g-h) Graphs depicting IC_50_ values on day 4 utilizing 3−0 and 2−0 scoring, respectively. i) Graph depicting LC_50_ values on day 4. Graphs correspond to values in Table 4.
(TIF)

S1 Table IT_50_ and LT_50_ values (2−0 scoring) for *C. elegans* on different anthelmintics (days). IT_50_ and LT_50_ values (from the unbiased 2−0 scoring system) for *C. elegans* on different anthelmintics are reported here for the L1 stage for pyrantel, nitazoxanide, ivermectin, albendazole, and mebendazole, and the L4 stage for mebendazole only. They are color-coded with the same color used for the same concentration on the line graphs (Figures 1b-6b for LT_50_ and S1–S6 Figs for IT_50_ values). U = undefined, N/A = not applicable because it was not performed.
(TIF)

S2 Table P-values for Two-Way ANOVA with Dunnett’s multiple comparisons tests for each drug tested (utilizing 3−0 and 2−0 motility data and 1−0 mortality data). P-values for the Two-Way ANOVA analyses and each factor’s percent of total variation are reported here. P-values for Dunnett’s multiple comparisons tests are also presented, comparing the experimental versus control for each day and drug. ns = not significant, while the asterisks denote the significance level (from * to ****). N/A = Not Applicable, utilized if the concentration was not tested for the given drug.
(TIF)
