# Correction: Mass drug administration approved and candidate anthelmintics beginning on larval stage 1 *Caenorhabditis elegans* are generally potent, suggesting a novel, pre-infective control for helminths

**DOI:** 10.1371/journal.pone.0355879

**Published:** 2026-08-11

**Authors:** Sam A. Attalla, Kathryn J. Isaac, Morgan Pfeffer, Justin R. Hockaday, Raegan Weatherly, Malia B. Asselin, Allison K. Lewis, Viveke Rai, William A. Huff Jr, Mason H. Long, Tashna Placide, Kala R. Pearce, Leopold N. Nkengbeza, Anna K. Thurman, Audrey L. Cox, Giselle Domingo Diaz, Jasmine Carter, Nathan Stein, Sam Fischer, Brian L. Ellis

S2 Table was uploaded incorrectly. Please see the correct S2 Table here.

## Supporting information

S2 Table P-values for Two-Way ANOVA with Dunnett’s multiple comparisons tests for each drug tested (utilizing 3 − 0 and 2 − 0 motility data and 1 − 0 mortality data). P-values for the Two-Way ANOVA analyses and each factor’s percent of total variation are reported here. P-values for Dunnett’s multiple comparisons tests are also presented, comparing the experimental versus control for each day and drug. ns = not significant, while the asterisks denote the significance level (from * to ****). N/A = Not Applicable, utilized if the concentration was not tested for the given drug.
(PDF)
