# Supplementary figures and images for: Mass drug administration approved and candidate anthelmintics beginning on larval stage 1 Caenorhabditis elegans are generally potent, suggesting a novel, pre-infective control for helminths
Source: PLoS One. 2026 Apr 17;21(4):e0346795. doi: 10.1371/journal.pone.0346795 (PMC13089895; doi:10.1371/journal.pone.0346795)

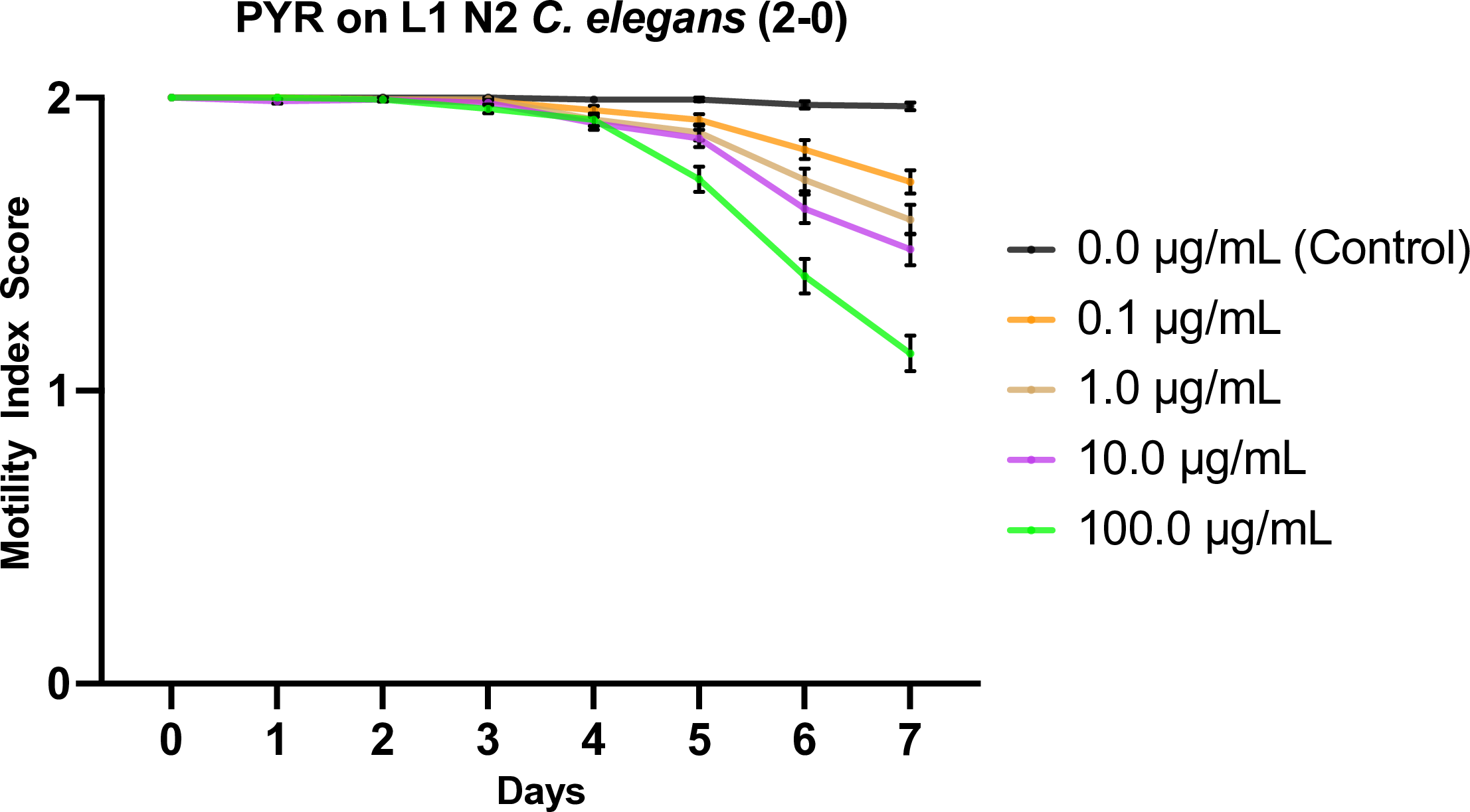

Supplement: S1 Fig — Graph of average sample health rating utilizing motility index scale (0–2). “2” represents a parasite with whole-body movements (observed without external stimulus) significantly slower than control no drug; “1” represents a parasite that was not moving on its own but moved when touched with a probe (tested at three different body locations); and 0 represents a worm that did not move even when prodded. (TIF) [file pone.0346795.s001.tif]

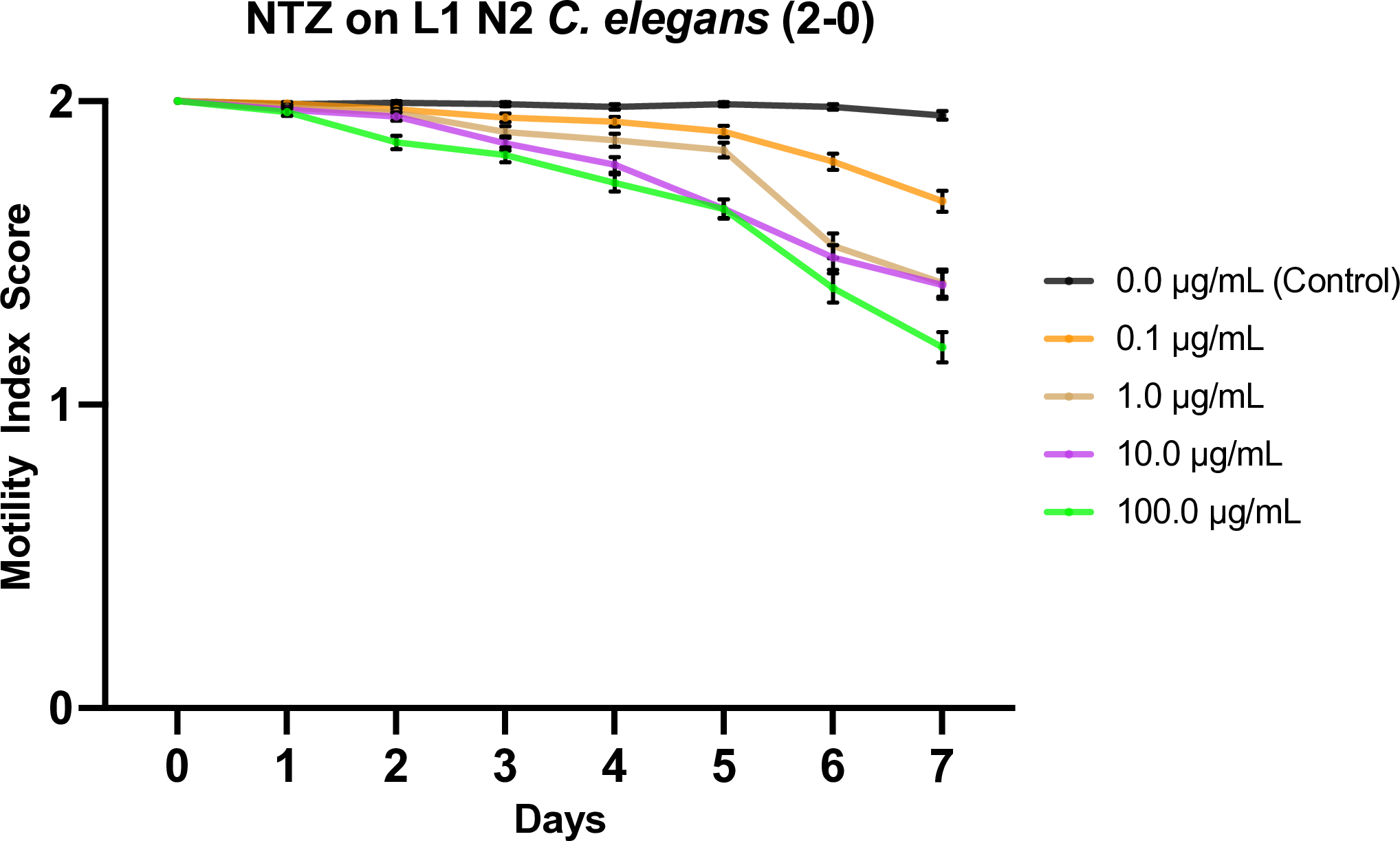

Supplement: S2 Fig — Graph of average sample health rating utilizing motility index scale (0–2). “2” represents a parasite with whole-body movements (observed without external stimulus) significantly slower than control no drug; “1” represents a parasite that was not moving on its own but moved when touched with a probe (tested at three different body locations); and 0 represents a worm that did not move even when prodded. (TIF) [file pone.0346795.s002.tif]

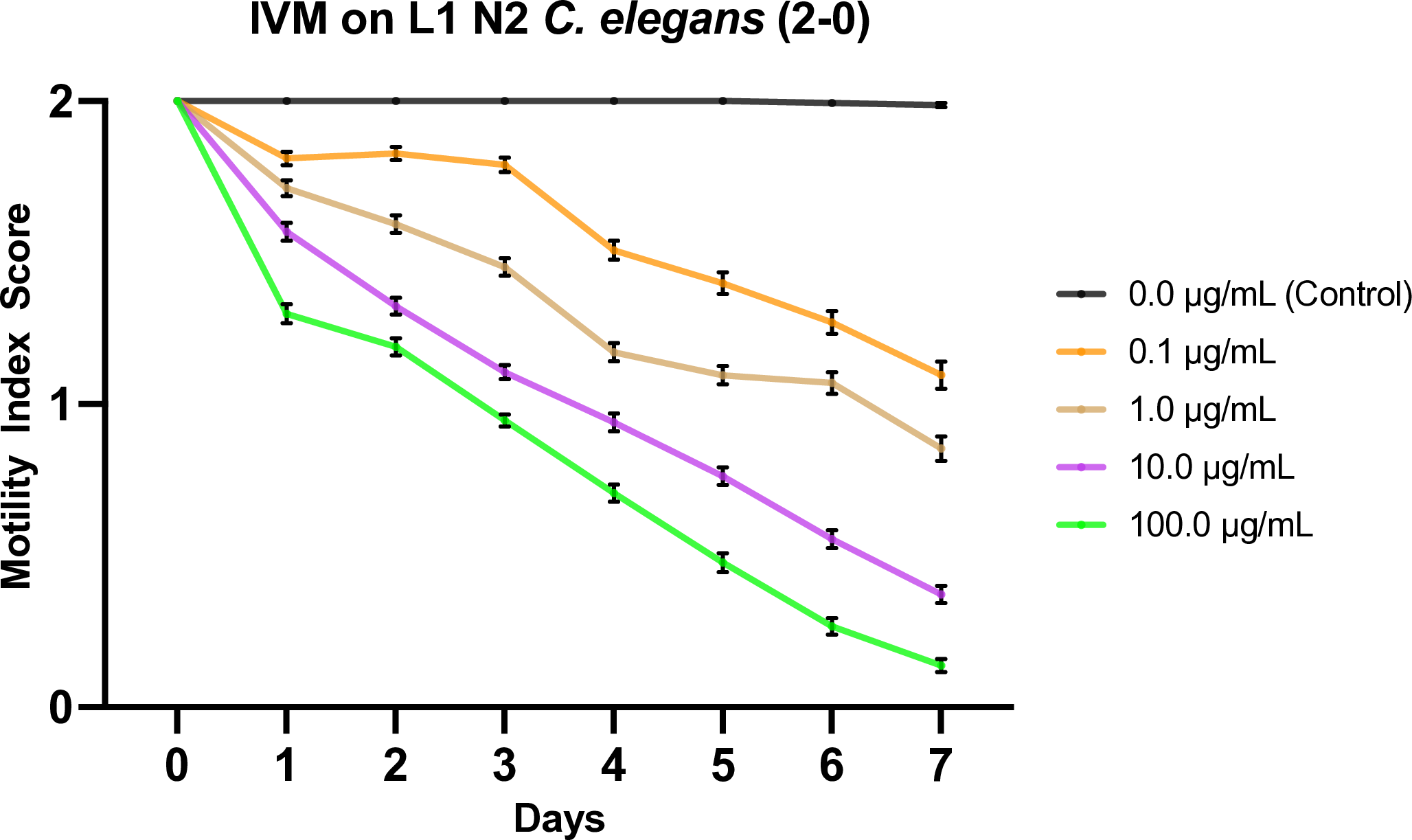

Supplement: S3 Fig — Graph of average sample health rating utilizing motility index scale (0–2). “2” represents a parasite with whole-body movements (observed without external stimulus) significantly slower than control no drug; “1” represents a parasite that was not moving on its own but moved when touched with a probe (tested at three different body locations); and 0 represents a worm that did not move even when prodded. (TIF) [file pone.0346795.s003.tif]

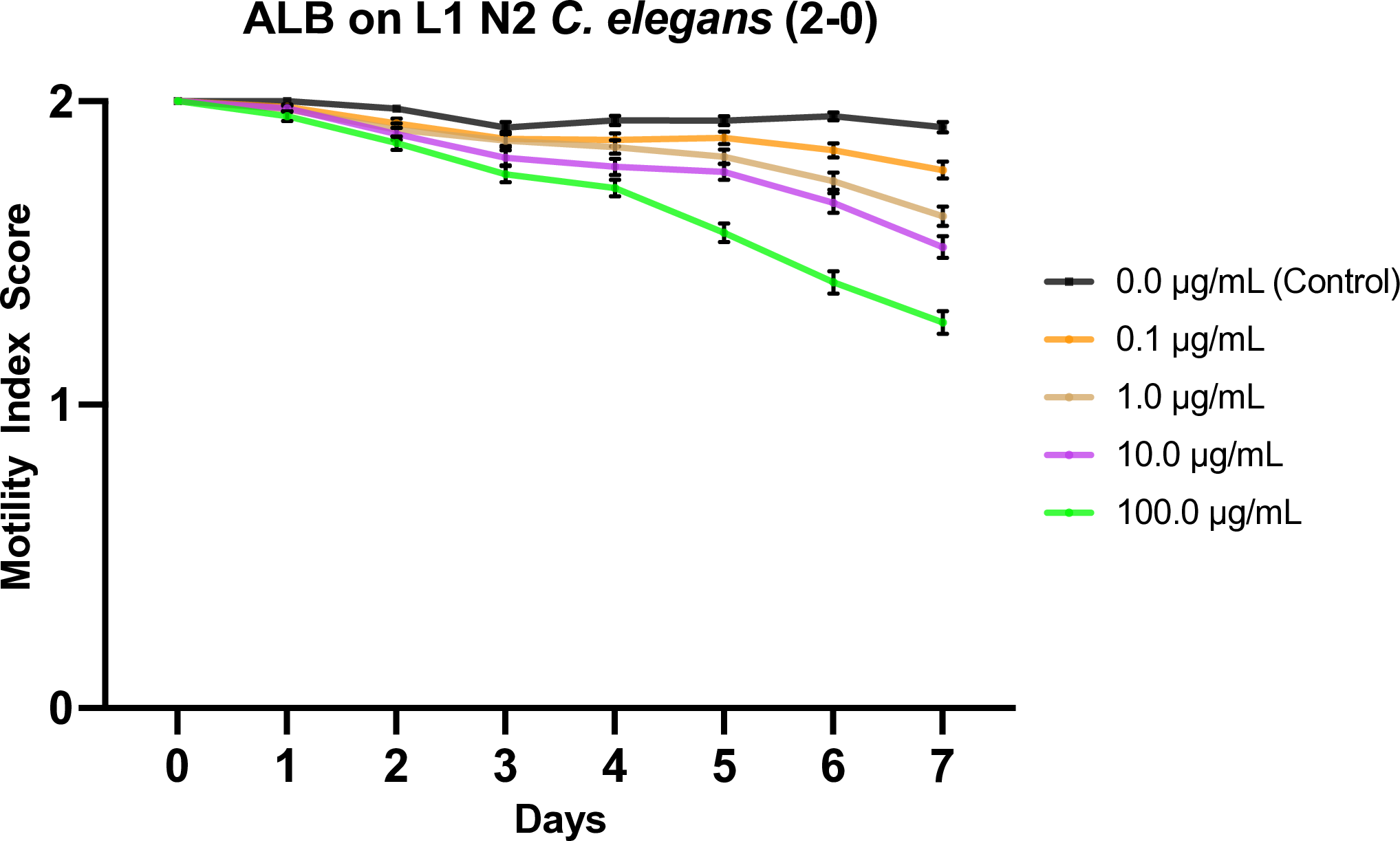

Supplement: S4 Fig — Graph of average sample health rating utilizing motility index scale (0–2). “2” represents a parasite with whole-body movements (observed without external stimulus) significantly slower than control no drug; “1” represents a parasite that was not moving on its own but moved when touched with a probe (tested at three different body locations); and 0 represents a worm that did not move even when prodded. (TIF) [file pone.0346795.s004.tif]

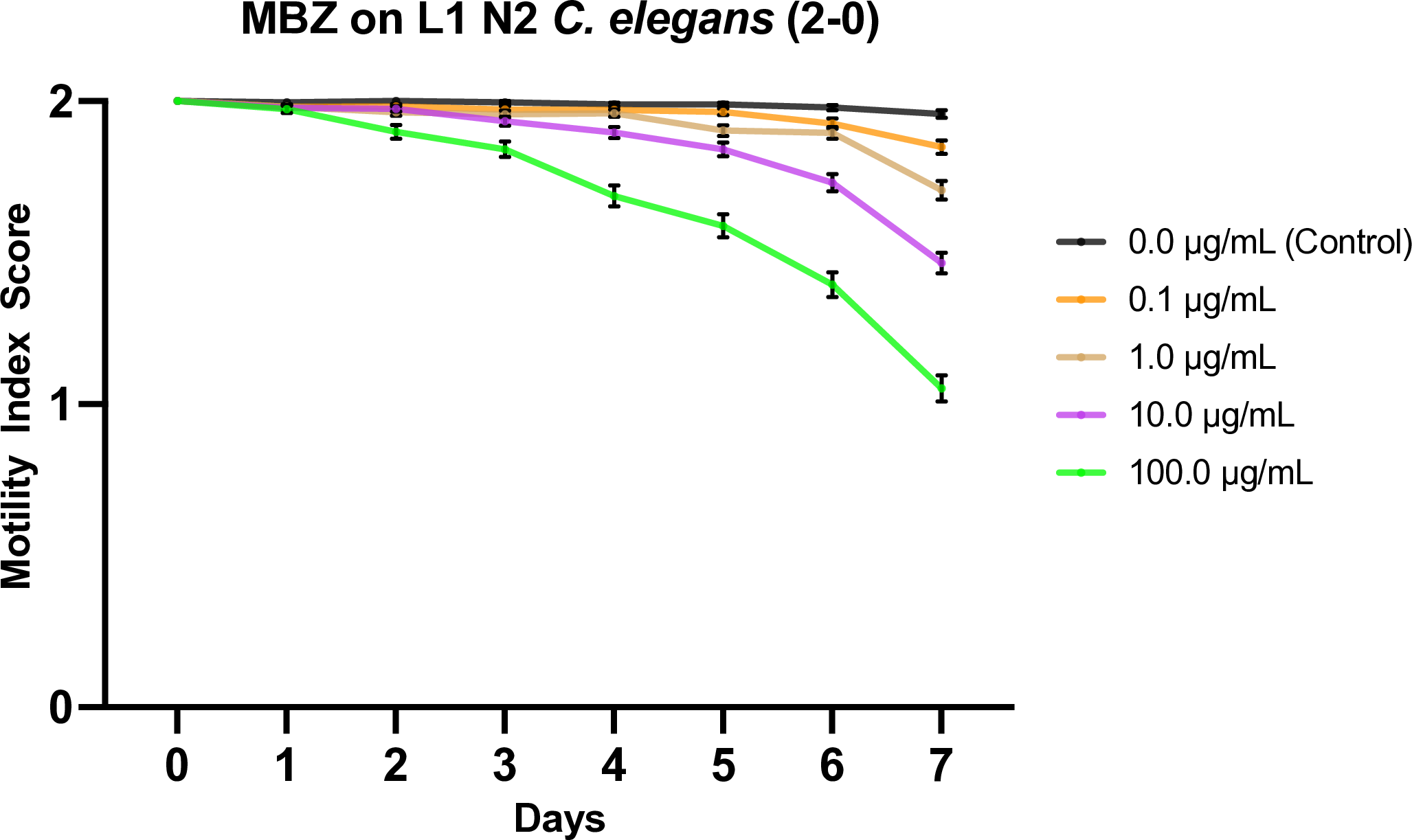

Supplement: S5 Fig — Graph of average sample health rating utilizing motility index scale (0–2). “2” represents a parasite with whole-body movements (observed without external stimulus) significantly slower than control no drug; “1” represents a parasite that was not moving on its own but moved when touched with a probe (tested at three different body locations); and 0 represents a worm that did not move even when prodded. (TIF) [file pone.0346795.s005.tif]

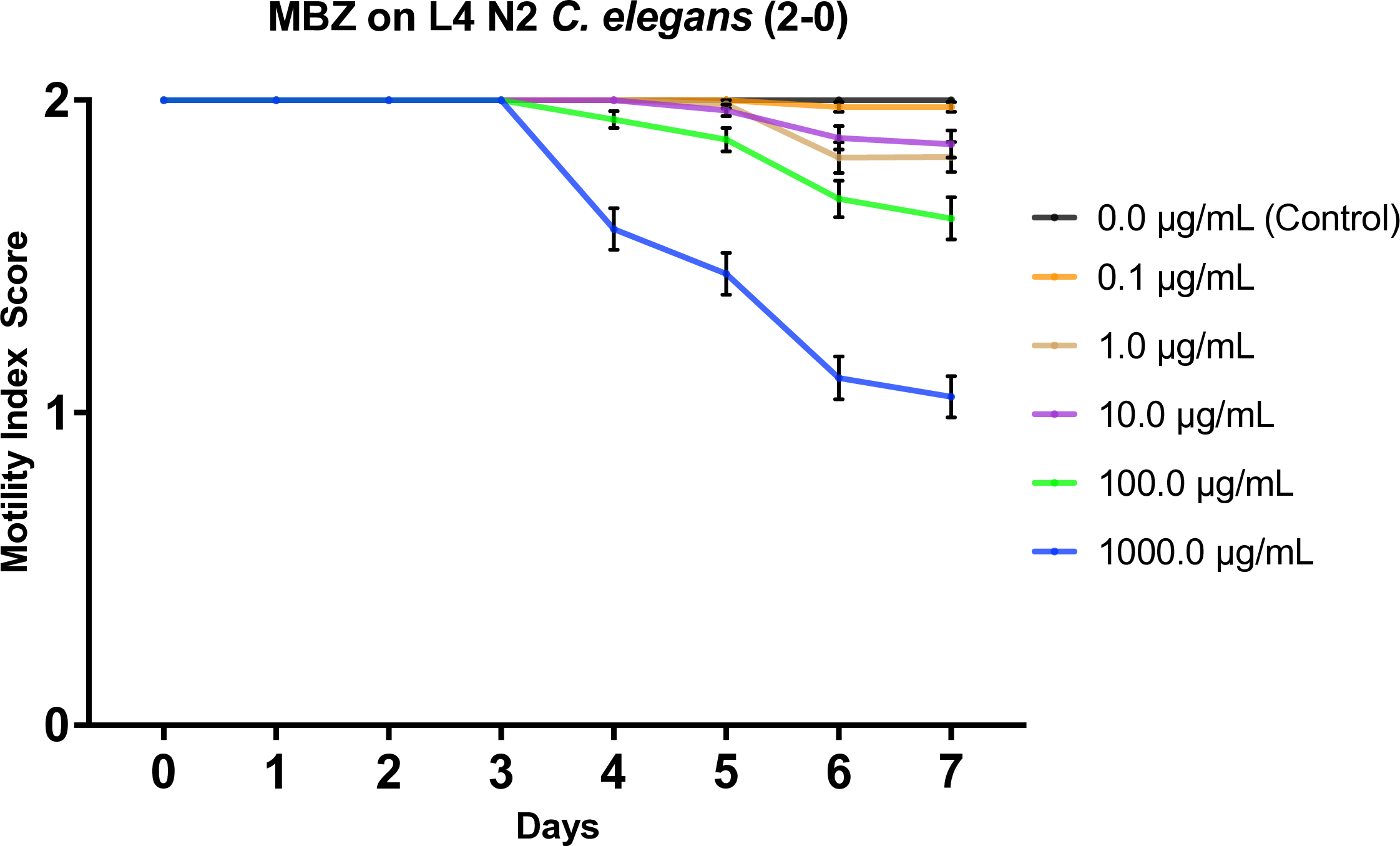

Supplement: S6 Fig — Graph of average sample health rating utilizing motility index scale (0–2). “2” represents a parasite with whole-body movements (observed without external stimulus) significantly slower than control no drug; “1” represents a parasite that was not moving on its own but moved when touched with a probe (tested at three different body locations); and 0 represents a worm that did not move even when prodded. (TIF) [file pone.0346795.s006.tif]

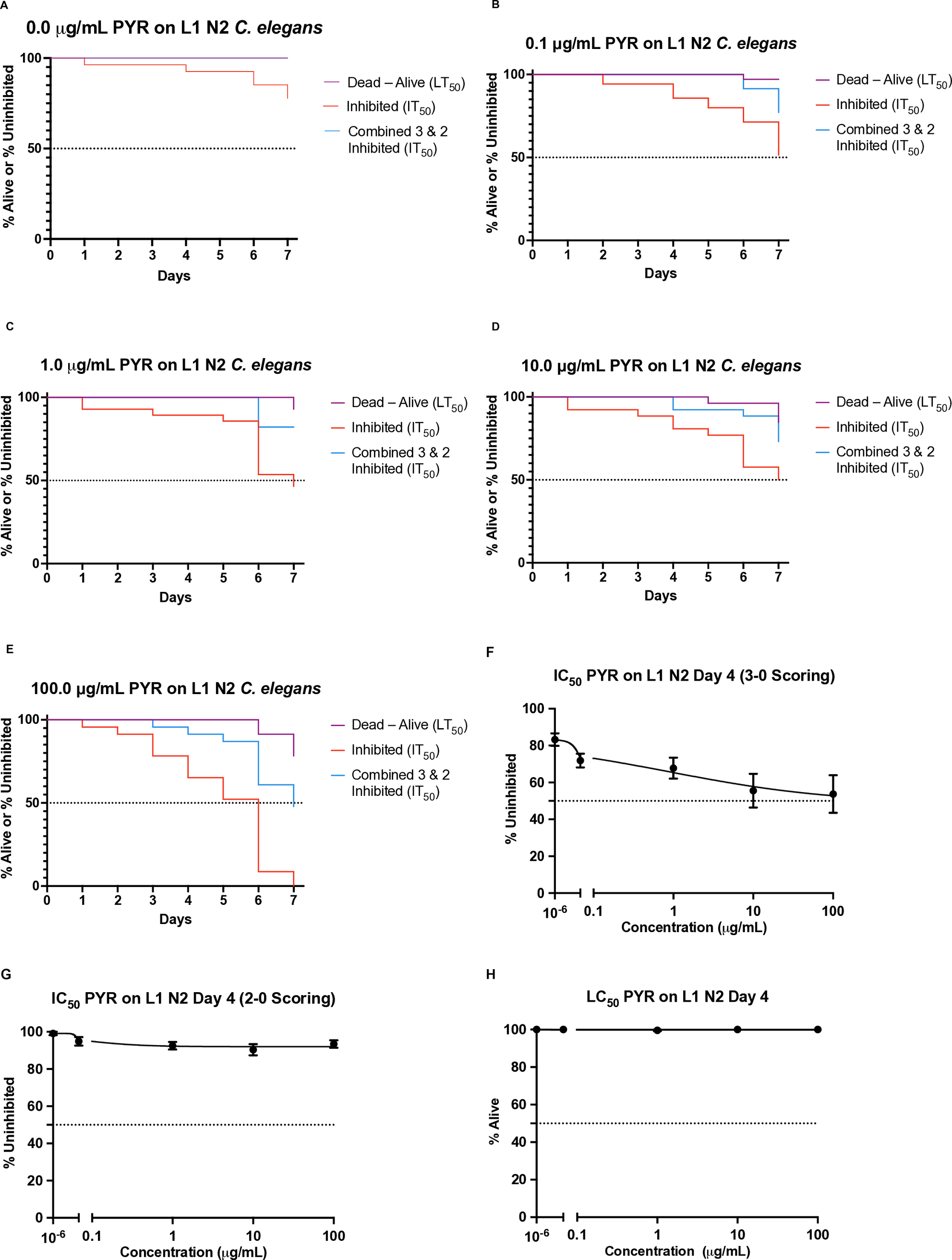

Supplement: S7 Fig — C. elegans. a-e) Graphs depicting LT50 (Purple), IT50 3−0 scoring (Orange), and combined 3 & 2 (2−0 scoring) IT50 (Blue) values for worms exposed to increasing concentrations of the drug. Graphs correspond to values in Table 3 (3−0 scoring) and S1 Table (2−0 scoring). f-g) Graphs depicting IC50 values on day 4 utilizing 3−0 and 2−0 scoring, respectively. h) Graph depicting LC50 values on day 4. Graphs correspond to values in Table 4. (TIF) [file pone.0346795.s007.tif]

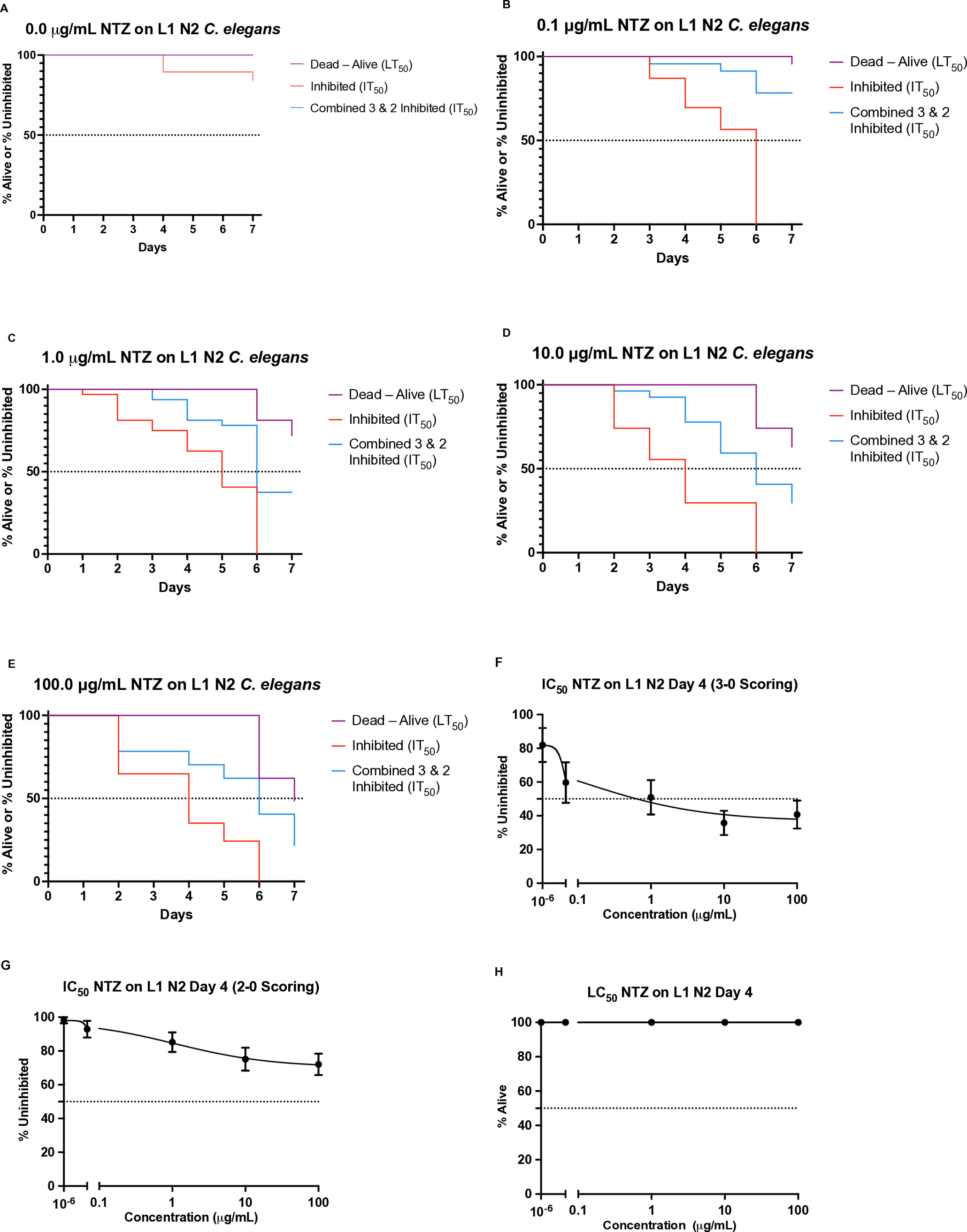

Supplement: S8 Fig — C. elegans. a-e) Graphs depicting LT50 (Purple), IT50 3−0 scoring (Orange), and combined 3 & 2 (2−0 scoring) IT50 (Blue) values for worms exposed to increasing concentrations of the drug. Graphs correspond to values in Table 3 (3−0 scoring) and S1 Table (2−0 scoring). f-g) Graphs depicting IC50 values on day 4 utilizing 3−0 and 2−0 scoring, respectively. h) Graph depicting LC50 values on day 4. Graphs correspond to values in Table 4. (TIF) [file pone.0346795.s008.tif]

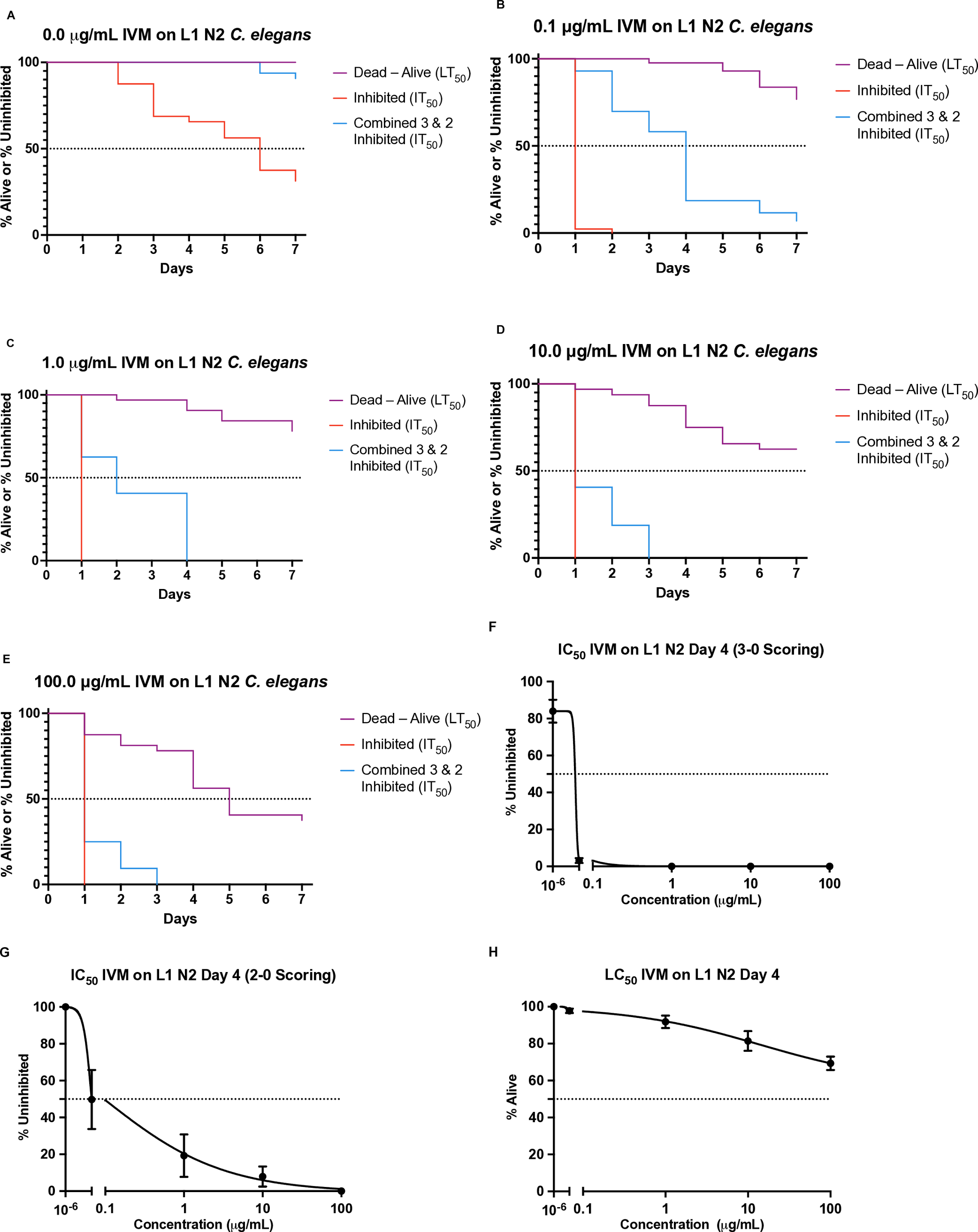

Supplement: S9 Fig — C. elegans. a-e) Graphs depicting LT50 (Purple), IT50 3−0 scoring (Orange), and combined 3 & 2 (2−0 scoring) IT50 (Blue) values for worms exposed to increasing concentrations of the drug. Graphs correspond to values in Table 3 (3−0 scoring) and S1 Table (2−0 scoring). f-g) Graphs depicting IC50 values on day 4 utilizing 3−0 and 2−0 scoring, respectively. h) Graph depicting LC50 values on day 4. Graphs correspond to values in Table 4. (TIF) [file pone.0346795.s009.tif]

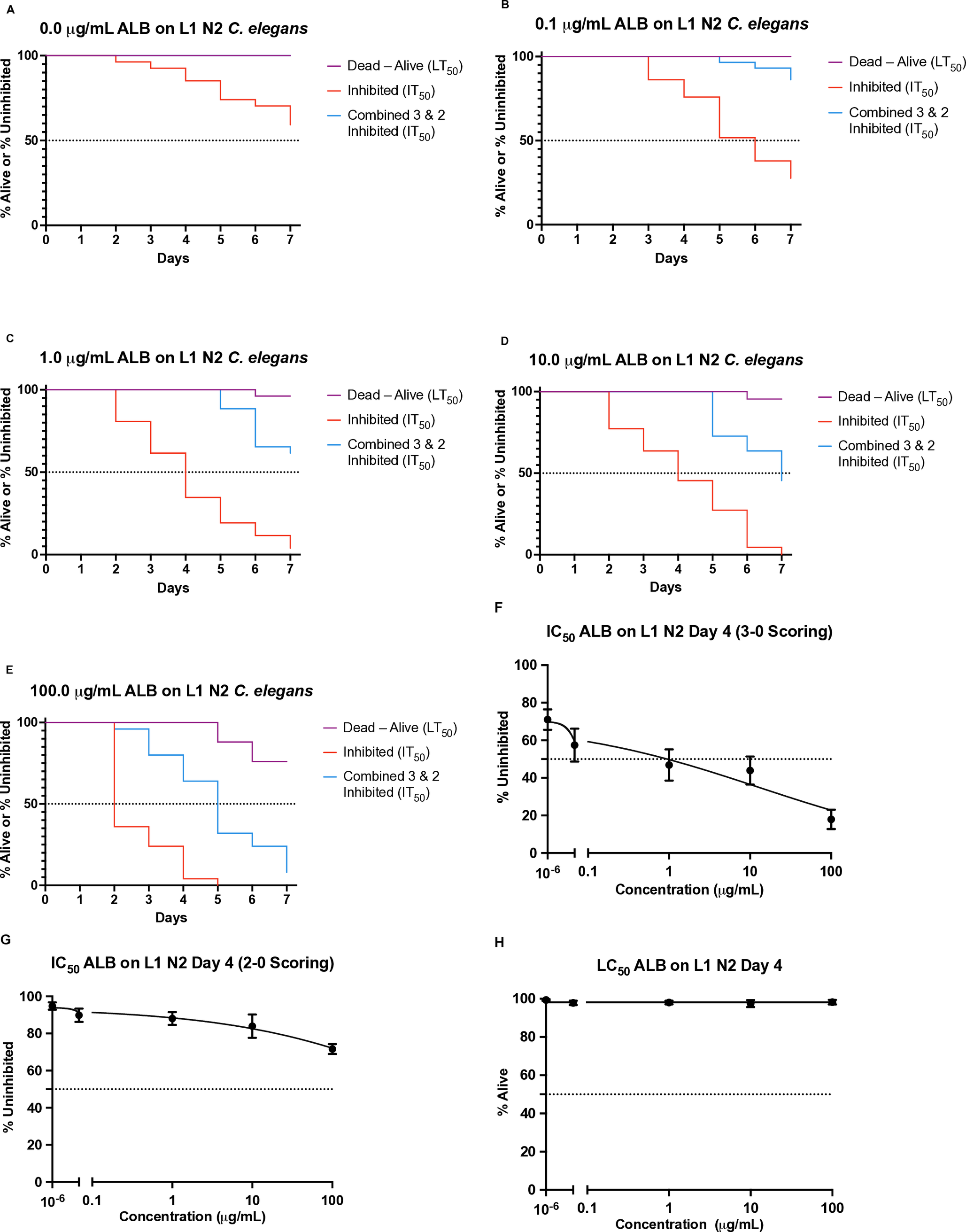

Supplement: S10 Fig — C. elegans. a-e) Graphs depicting LT50 (Purple), IT50 3−0 scoring (Orange), and combined 3 & 2 (2−0 scoring) IT50 (Blue) values for worms exposed to increasing concentrations of the drug. Graphs correspond to values in Table 3 (3−0 scoring) and S1 Table (2−0 scoring). f-g) Graphs depicting IC50 values on day 4 utilizing 3−0 and 2−0 scoring, respectively. h) Graph depicting LC50 values on day 4. Graphs correspond to values in Table 4. (TIF) [file pone.0346795.s010.tif]

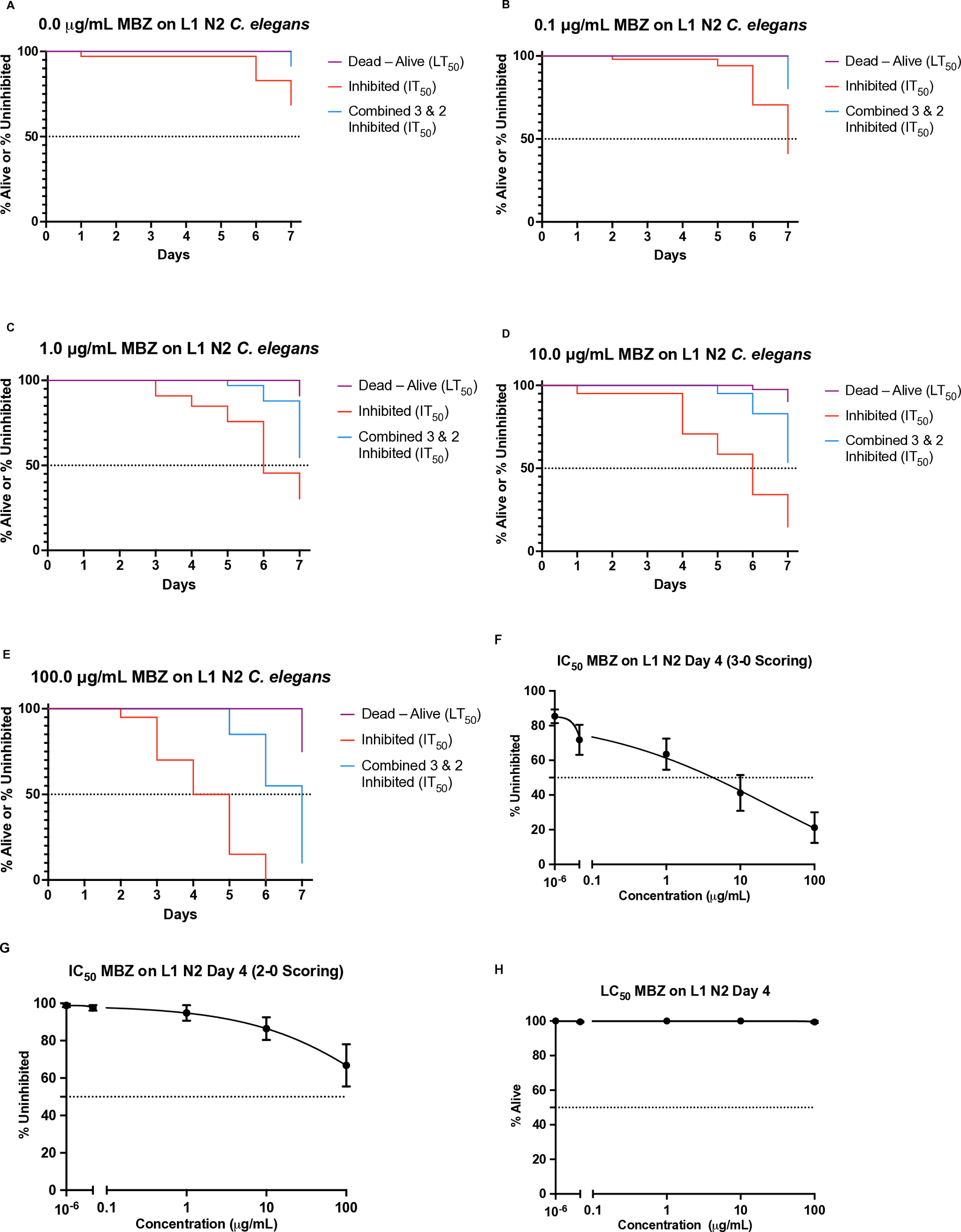

Supplement: S11 Fig — C. elegans. a-e) Graphs depicting LT50 (Purple), IT50 3−0 scoring (Orange), and combined 3 & 2 (2−0 scoring) IT50 (Blue) values for worms exposed to increasing concentrations of the drug. Graphs correspond to values in Table 3 (3−0 scoring) and S1 Table (2−0 scoring). f-g) Graphs depicting IC50 values on day 4 utilizing 3−0 and 2−0 scoring, respectively. h) Graph depicting LC50 values on day 4. Graphs correspond to values in Table 4. (TIF) [file pone.0346795.s011.tif]

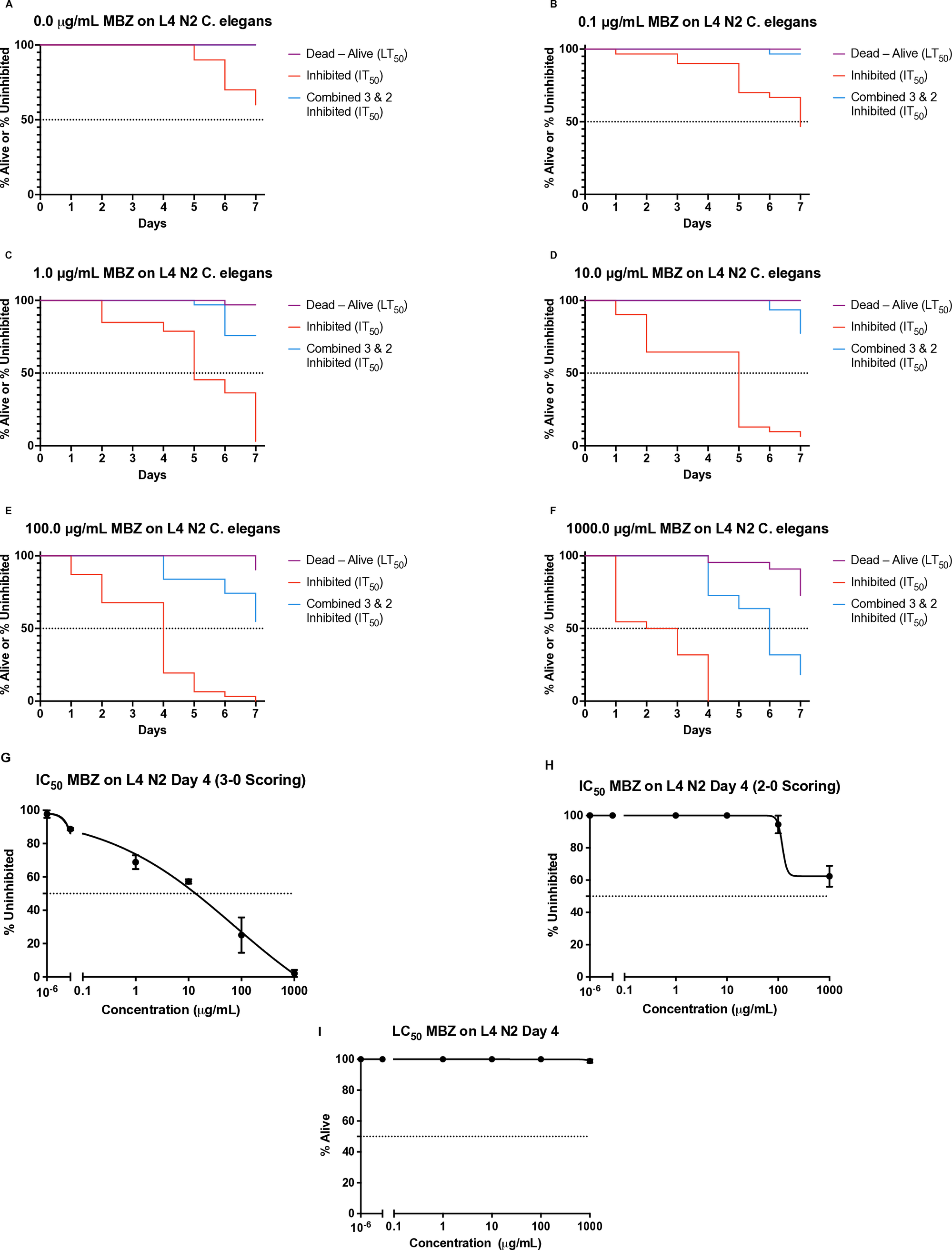

Supplement: S12 Fig — C. elegans. a-f) Graphs depicting LT50 (Purple), IT50 3−0 scoring (Orange), and combined 3 & 2 (2−0 scoring) IT50 (Blue) values for worms exposed to increasing concentrations of the drug. Graphs correspond to values in Table 3 (3−0 scoring) and S1 Table (2−0 scoring). g-h) Graphs depicting IC50 values on day 4 utilizing 3−0 and 2−0 scoring, respectively. i) Graph depicting LC50 values on day 4. Graphs correspond to values in Table 4. (TIF) [file pone.0346795.s012.tif]

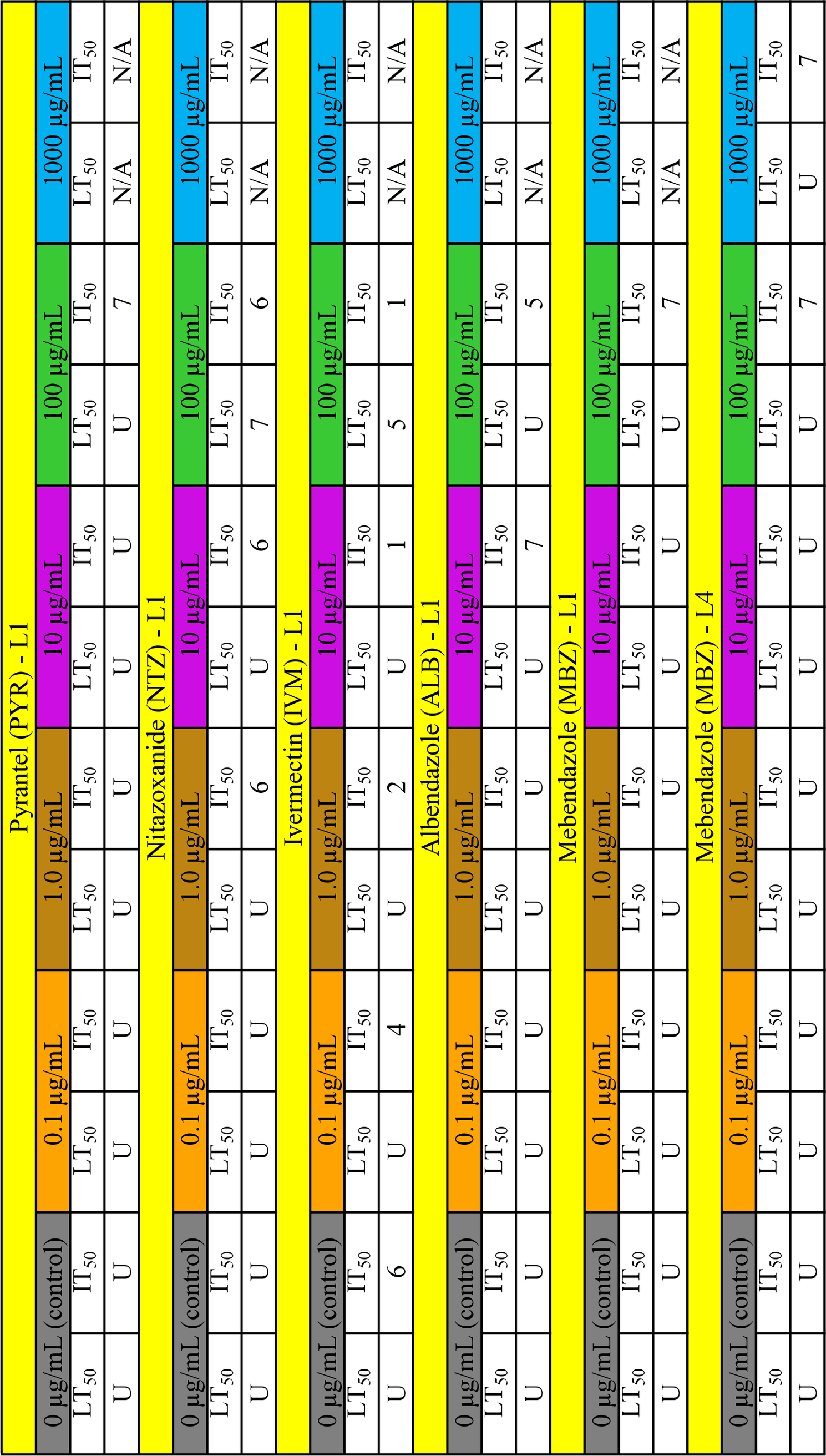

Supplement: S1 Table — IT50 and LT50 values (from the unbiased 2−0 scoring system) for C. elegans on different anthelmintics are reported here for the L1 stage for pyrantel, nitazoxanide, ivermectin, albendazole, and mebendazole, and the L4 stage for mebendazole only. They are color-coded with the same color used for the same concentration on the line graphs (Figures 1b-6b for LT50 and S1–S6 Figs for IT50 values). U = undefined, N/A = not applicable because it was not performed. (TIF) [file pone.0346795.s013.tif]

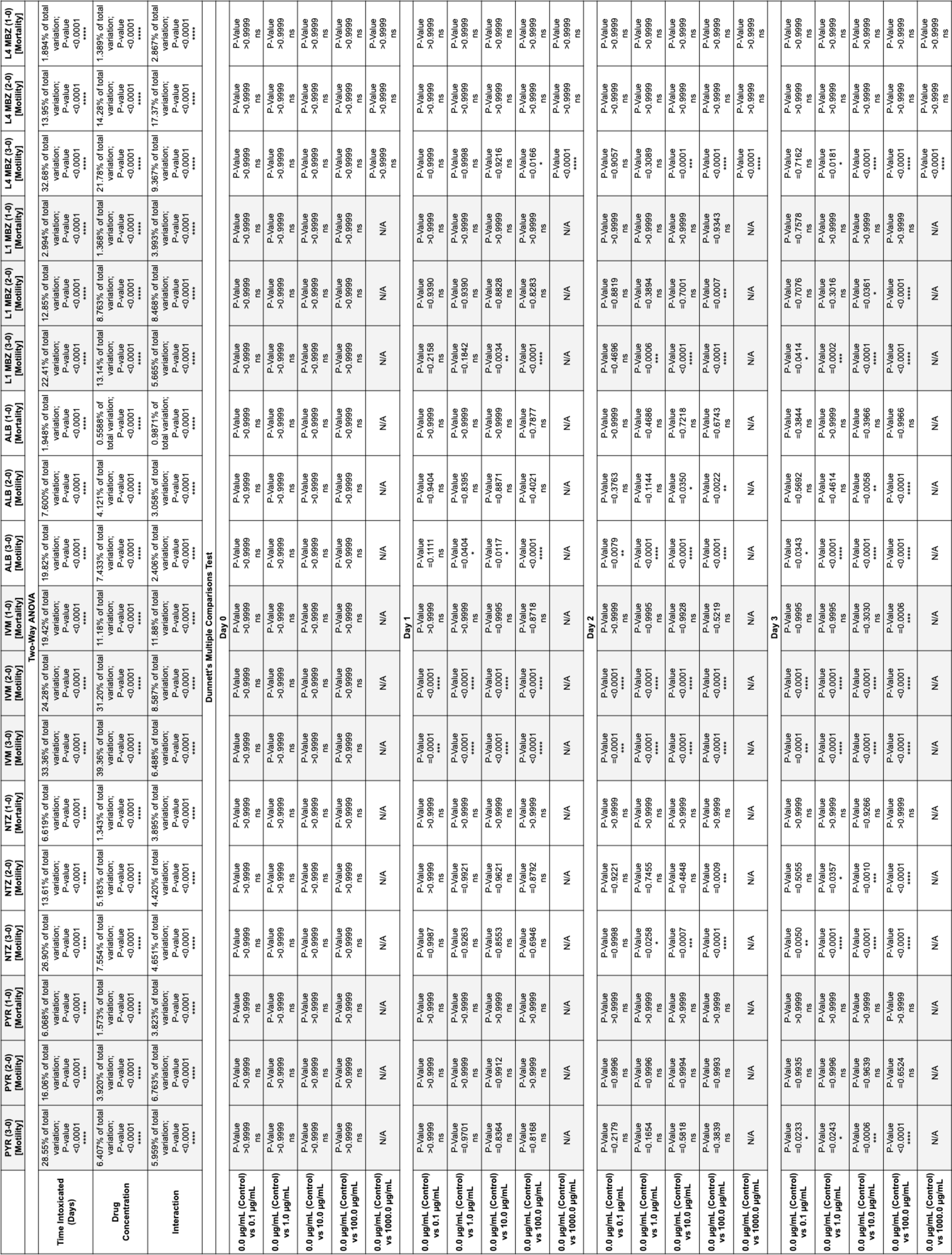

Supplement: S2 Table — P-values for the Two-Way ANOVA analyses and each factor’s percent of total variation are reported here. P-values for Dunnett’s multiple comparisons tests are also presented, comparing the experimental versus control for each day and drug. ns = not significant, while the asterisks denote the significance level (from * to ****). N/A = Not Applicable, utilized if the concentration was not tested for the given drug. (TIF) [file pone.0346795.s014.tif]
