# Supplementary material for: Correction: Mass drug administration approved and candidate anthelmintics beginning on larval stage 1 Caenorhabditis elegans are generally potent, suggesting a novel, pre-infective control for helminths
Source: PLoS One. 2026 Aug 11;21(8):e0355879. doi: 10.1371/journal.pone.0355879 (PMC13460568; doi:10.1371/journal.pone.0355879)
Supplement: Supplementary file 1 [file pone.0355879.s001.pdf]

	PYR (3-0) [Motility]	PYR (2-0) [Motility]	PYR (1-0) [Mortality]	NTZ (3-0) [Motility]	NTZ (2-0) [Motility]	NTZ (1-0) [Mortality]	IVM (3-0) [Motility]	IVM (2-0) [Motility]	IVM (1-0) [Mortality]	ALB (3-0) [Motility]	ALB (2-0) [Motility]	ALB (1-0) [Mortality]	L1 MBZ (3-0) [Motility]	L1 MBZ (2-0) [Motility]	L1 MBZ (1-0) [Mortality]	L4 MBZ (3-0) [Motility]	L4 MBZ (2-0) [Motility]	L4 MBZ (1-0) [Mortality]
**Two-Way ANOVA**																		
**Time Intoxicated (Days)**	28.55% of total variation; P-value <0.0001 ****	16.06% of total variation; P-value <0.0001 ****	6.068% of total variation; P-value <0.0001 ****	26.90% of total variation; P-value <0.0001 ****	13.61% of total variation; P-value <0.0001 ****	6.619% of total variation; P-value <0.0001 ****	33.36% of total variation; P-value <0.0001 ****	24.28% of total variation; P-value <0.0001 ****	19.42% of total variation; P-value <0.0001 ****	19.82% of total variation; P-value <0.0001 ****	7.600% of total variation; P-value <0.0001 ****	1.948% of total variation; P-value <0.0001 ****	22.41% of total variation; P-value <0.0001 ****	12.85% of total variation; P-value <0.0001 ****	2.994% of total variation; P-value <0.0001 ****	32.68% of total variation; P-value <0.0001 ****	13.95% of total variation; P-value <0.0001 ****	1.894% of total variation; P-value <0.0001 ****
**Drug Concentration**	6.407% of total variation; P-value <0.0001 ****	3.920% of total variation; P-value <0.0001 ****	1.573% of total variation; P-value <0.0001 ****	7.554% of total variation; P-value <0.0001 ****	5.183% of total variation; P-value <0.0001 ****	1.343% of total variation; P-value <0.0001 ****	39.36% of total variation; P-value <0.0001 ****	31.20% of total variation; P-value <0.0001 ****	11.18% of total variation; P-value <0.0001 ****	7.433% of total variation; P-value <0.0001 ****	4.121% of total variation; P-value <0.0001 ****	0.5588% of total variation; P-value <0.0001 ****	13.14% of total variation; P-value <0.0001 ****	8.763% of total variation; P-value <0.0001 ****	1.368% of total variation; P-value <0.0001 ****	21.78% of total variation; P-value <0.0001 ****	14.28% of total variation; P-value <0.0001 ****	1.389% of total variation; P-value <0.0001 ****
**Interaction**	5.959% of total variation; P-value <0.0001 ****	6.763% of total variation; P-value <0.0001 ****	3.823% of total variation; P-value <0.0001 ****	4.651% of total variation; P-value <0.0001 ****	4.420% of total variation; P-value <0.0001 ****	3.895% of total variation; P-value <0.0001 ****	6.488% of total variation; P-value <0.0001 ****	8.587% of total variation; P-value <0.0001 ****	11.88% of total variation; P-value <0.0001 ****	2.406% of total variation; P-value <0.0001 ****	3.058% of total variation; P-value <0.0001 ****	0.9871% of total variation; P-value <0.0001 ****	5.665% of total variation; P-value <0.0001 ****	8.468% of total variation; P-value <0.0001 ****	3.993% of total variation; P-value <0.0001 ****	9.367% of total variation; P-value <0.0001 ****	17.37% of total variation; P-value <0.0001 ****	2.867% of total variation; P-value <0.0001 ****
**Dunnett's Multiple Comparisons Test**																		
**Day 0**																		
**0.0 µg/mL (Control) vs 0.1 µg/mL**	P-Value >0.9999 ns	P-Value >0.9999 ns	P-Value >0.9999 ns	P-Value >0.9999 ns	P-Value >0.9999 ns	P-Value >0.9999 ns	P-Value >0.9999 ns	P-Value >0.9999 ns	P-Value >0.9999 ns	P-Value >0.9999 ns	P-Value >0.9999 ns	P-Value >0.9999 ns	P-Value >0.9999 ns	P-Value >0.9999 ns	P-Value >0.9999 ns	P-Value >0.9999 ns	P-Value >0.9999 ns	P-Value >0.9999 ns
**0.0 µg/mL (Control) vs 1.0 µg/mL**	P-Value >0.9999 ns	P-Value >0.9999 ns	P-Value >0.9999 ns	P-Value >0.9999 ns	P-Value >0.9999 ns	P-Value >0.9999 ns	P-Value >0.9999 ns	P-Value >0.9999 ns	P-Value >0.9999 ns	P-Value >0.9999 ns	P-Value >0.9999 ns	P-Value >0.9999 ns	P-Value >0.9999 ns	P-Value >0.9999 ns	P-Value >0.9999 ns	P-Value >0.9999 ns	P-Value >0.9999 ns	P-Value >0.9999 ns
**0.0 µg/mL (Control) vs 10.0 µg/mL**	P-Value >0.9999 ns	P-Value >0.9999 ns	P-Value >0.9999 ns	P-Value >0.9999 ns	P-Value >0.9999 ns	P-Value >0.9999 ns	P-Value >0.9999 ns	P-Value >0.9999 ns	P-Value >0.9999 ns	P-Value >0.9999 ns	P-Value >0.9999 ns	P-Value >0.9999 ns	P-Value >0.9999 ns	P-Value >0.9999 ns	P-Value >0.9999 ns	P-Value >0.9999 ns	P-Value >0.9999 ns	P-Value >0.9999 ns
**0.0 µg/mL (Control) vs 100.0 µg/mL**	P-Value >0.9999 ns	P-Value >0.9999 ns	P-Value >0.9999 ns	P-Value >0.9999 ns	P-Value >0.9999 ns	P-Value >0.9999 ns	P-Value >0.9999 ns	P-Value >0.9999 ns	P-Value >0.9999 ns	P-Value >0.9999 ns	P-Value >0.9999 ns	P-Value >0.9999 ns	P-Value >0.9999 ns	P-Value >0.9999 ns	P-Value >0.9999 ns	P-Value >0.9999 ns	P-Value >0.9999 ns	P-Value >0.9999 ns
**0.0 µg/mL (Control) vs 1000.0 µg/mL**	N/A	N/A	N/A	N/A	N/A	N/A	N/A	N/A	N/A	N/A	N/A	N/A	N/A	N/A	N/A	P-Value >0.9999 ns	P-Value >0.9999 ns	P-Value >0.9999 ns
**Day 1**																		
**0.0 µg/mL (Control) vs 0.1 µg/mL**	P-Value >0.9999 ns	P-Value >0.9999 ns	P-Value >0.9999 ns	P-Value =0.9987 ns	P-Value >0.9999 ns	P-Value >0.9999 ns	P-Value =0.0001 ***	P-Value <0.0001 ****	P-Value >0.9999 ns	P-Value =0.1111 ns	P-Value =0.9404 ns	P-Value >0.9999 ns	P-Value =0.2158 ns	P-Value =0.9390 ns	P-Value >0.9999 ns	P-Value =0.9999 ns	P-Value >0.9999 ns	P-Value >0.9999 ns
**0.0 µg/mL (Control) vs 1.0 µg/mL**	P-Value =0.9701 ns	P-Value >0.9999 ns	P-Value >0.9999 ns	P-Value =0.9263 ns	P-Value =0.9921 ns	P-Value >0.9999 ns	P-Value <0.0001 ****	P-Value <0.0001 ****	P-Value >0.9999 ns	P-Value =0.0404 *	P-Value =0.8395 ns	P-Value >0.9999 ns	P-Value =0.1842 ns	P-Value =0.9390 ns	P-Value >0.9999 ns	P-Value =0.9998 ns	P-Value >0.9999 ns	P-Value >0.9999 ns
**0.0 µg/mL (Control) vs 10.0 µg/mL**	P-Value =0.8364 ns	P-Value =0.9912 ns	P-Value >0.9999 ns	P-Value =0.8553 ns	P-Value =0.9621 ns	P-Value >0.9999 ns	P-Value <0.0001 ****	P-Value <0.0001 ****	P-Value =0.9995 ns	P-Value =0.0117 *	P-Value =0.8871 ns	P-Value >0.9999 ns	P-Value =0.0034 **	P-Value =0.8828 ns	P-Value >0.9999 ns	P-Value =0.9216 ns	P-Value >0.9999 ns	P-Value >0.9999 ns
**0.0 µg/mL (Control) vs 100.0 µg/mL**	P-Value =0.8168 ns	P-Value >0.9999 ns	P-Value >0.9999 ns	P-Value =0.6946 ns	P-Value =0.8792 ns	P-Value >0.9999 ns	P-Value <0.0001 ****	P-Value <0.0001 ****	P-Value =0.8718 ns	P-Value <0.0001 ****	P-Value =0.4021 ns	P-Value =0.7877 ns	P-Value <0.0001 ****	P-Value =0.8283 ns	P-Value >0.9999 ns	P-Value =0.0166 *	P-Value >0.9999 ns	P-Value >0.9999 ns
**0.0 µg/mL (Control) vs 1000.0 µg/mL**	N/A	N/A	N/A	N/A	N/A	N/A	N/A	N/A	N/A	N/A	N/A	N/A	N/A	N/A	N/A	P-Value <0.0001 ****	P-Value >0.9999 ns	P-Value >0.9999 ns
**Day 2**																		
**0.0 µg/mL (Control) vs 0.1 µg/mL**	P-Value =0.2179 ns	P-Value =0.9996 ns	P-Value >0.9999 ns	P-Value =0.9998 ns	P-Value =0.9221 ns	P-Value >0.9999 ns	P-Value =0.0001 ***	P-Value <0.0001 ****	P-Value >0.9999 ns	P-Value =0.0079 **	P-Value =0.3763 ns	P-Value >0.9999 ns	P-Value =0.4696 ns	P-Value =0.8819 ns	P-Value >0.9999 ns	P-Value =0.9057 ns	P-Value >0.9999 ns	P-Value >0.9999 ns
**0.0 µg/mL (Control) vs 1.0 µg/mL**	P-Value =0.1654 ns	P-Value =0.9996 ns	P-Value >0.9999 ns	P-Value =0.0258 *	P-Value =0.7455 ns	P-Value >0.9999 ns	P-Value <0.0001 ****	P-Value <0.0001 ****	P-Value =0.9995 ns	P-Value <0.0001 ****	P-Value =0.1144 ns	P-Value =0.4686 ns	P-Value =0.0006 ***	P-Value =0.3894 ns	P-Value >0.9999 ns	P-Value =0.3089 ns	P-Value >0.9999 ns	P-Value >0.9999 ns
**0.0 µg/mL (Control) vs 10.0 µg/mL**	P-Value =0.5818 ns	P-Value =0.9994 ns	P-Value >0.9999 ns	P-Value =0.0007 ***	P-Value =0.4848 ns	P-Value >0.9999 ns	P-Value <0.0001 ****	P-Value <0.0001 ****	P-Value =0.9928 ns	P-Value <0.0001 ****	P-Value =0.0350 *	P-Value =0.7218 ns	P-Value <0.0001 ****	P-Value =0.7001 ns	P-Value >0.9999 ns	P-Value =0.0001 ***	P-Value >0.9999 ns	P-Value >0.9999 ns
**0.0 µg/mL (Control) vs 100.0 µg/mL**	P-Value =0.3839 ns	P-Value =0.9993 ns	P-Value >0.9999 ns	P-Value <0.0001 ****	P-Value =0.0009 ***	P-Value >0.9999 ns	P-Value <0.0001 ****	P-Value <0.0001 ****	P-Value =0.5219 ns	P-Value <0.0001 ****	P-Value =0.0022 **	P-Value =0.6743 ns	P-Value <0.0001 ****	P-Value =0.0007 ***	P-Value =0.9343 ns	P-Value <0.0001 ****	P-Value >0.9999 ns	P-Value >0.9999 ns
**0.0 µg/mL (Control) vs 1000.0 µg/mL**	N/A	N/A	N/A	N/A	N/A	N/A	N/A	N/A	N/A	N/A	N/A	N/A	N/A	N/A	N/A	P-Value <0.0001 ****	P-Value >0.9999 ns	P-Value >0.9999 ns
**Day 3**																		
**0.0 µg/mL (Control) vs 0.1 µg/mL**	P-Value =0.0233 *	P-Value =0.9935 ns	P-Value >0.9999 ns	P-Value =0.0050 **	P-Value =0.5055 ns	P-Value >0.9999 ns	P-Value =0.0001 ***	P-Value <0.0001 ****	P-Value =0.9995 ns	P-Value =0.0343 *	P-Value =0.5692 ns	P-Value =0.3844 ns	P-Value =0.0414 *	P-Value =0.7076 ns	P-Value =0.7578 ns	P-Value =0.7162 ns	P-Value >0.9999 ns	P-Value >0.9999 ns
**0.0 µg/mL (Control) vs 1.0 µg/mL**	P-Value =0.0243 *	P-Value =0.9996 ns	P-Value >0.9999 ns	P-Value <0.0001 ****	P-Value =0.0357 *	P-Value >0.9999 ns	P-Value <0.0001 ****	P-Value <0.0001 ****	P-Value =0.9995 ns	P-Value <0.0001 ****	P-Value =0.4614 ns	P-Value >0.9999 ns	P-Value =0.0002 ***	P-Value =0.3016 ns	P-Value >0.9999 ns	P-Value =0.0181 *	P-Value >0.9999 ns	P-Value >0.9999 ns
**0.0 µg/mL (Control) vs 10.0 µg/mL**	P-Value =0.0006 ***	P-Value =0.9639 ns	P-Value >0.9999 ns	P-Value <0.0001 ****	P-Value =0.0010 ***	P-Value =0.9266 ns	P-Value <0.0001 ****	P-Value <0.0001 ****	P-Value =0.3030 ns	P-Value <0.0001 ****	P-Value =0.0058 **	P-Value =0.3966 ns	P-Value <0.0001 ****	P-Value =0.0361 *	P-Value >0.9999 ns	P-Value <0.0001 ****	P-Value >0.9999 ns	P-Value >0.9999 ns
**0.0 µg/mL (Control) vs 100.0 µg/mL**	P-Value <0.0001 ****	P-Value =0.6524 ns	P-Value >0.9999 ns	P-Value <0.0001 ****	P-Value <0.0001 ****	P-Value >0.9999 ns	P-Value <0.0001 ****	P-Value <0.0001 ****	P-Value =0.0006 ***	P-Value <0.0001 ****	P-Value <0.0001 ****	P-Value =0.9966 ns	P-Value <0.0001 ****	P-Value <0.0001 ****	P-Value >0.9999 ns	P-Value <0.0001 ****	P-Value >0.9999 ns	P-Value >0.9999 ns
**0.0 µg/mL (Control) vs 1000.0 µg/mL**	N/A	N/A	N/A	N/A	N/A	N/A	N/A	N/A	N/A	N/A	N/A	N/A	N/A	N/A	N/A	P-Value <0.0001 ****	P-Value >0.9999 ns	P-Value >0.9999 ns

**Day 4**																		
**0.0 µg/mL (Control) vs 0.1 µg/mL**	P-Value =0.0405 *	P-Value =0.6438 ns	P-Value >0.9999 ns	P-Value <0.0001 ****	P-Value =0.4389 ns	P-Value >0.9999 ns	P-Value =0.0001 ***	P-Value <0.0001 ****	P-Value =0.7381 ns	P-Value =0.0005 ***	P-Value =0.1161 ns	P-Value =0.6785 ns	P-Value =0.0674 ns	P-Value =0.8810 ns	P-Value =0.9719 ns	P-Value =0.4447 ns	P-Value >0.9999 ns	P-Value >0.9999 ns
**0.0 µg/mL (Control) vs 1.0 µg/mL**	P-Value =0.0001 ***	P-Value =0.1337 ns	P-Value =0.9870 ns	P-Value <0.0001 ****	P-Value =0.0081 **	P-Value >0.9999 ns	P-Value <0.0001 ****	P-Value <0.0001 ****	P-Value =0.0025 **	P-Value <0.0001 ****	P-Value =0.0169 *	P-Value =0.8178 ns	P-Value =0.0001 ***	P-Value =0.5809 ns	P-Value >0.9999 ns	P-Value <0.0001 ****	P-Value >0.9999 ns	P-Value >0.9999 ns
**0.0 µg/mL (Control) vs 10.0 µg/mL**	P-Value <0.0001 ****	P-Value =0.0608 ns	P-Value >0.9999 ns	P-Value <0.0001 ****	P-Value <0.0001 ****	P-Value >0.9999 ns	P-Value <0.0001 ****	P-Value <0.0001 ****	P-Value <0.0001 ****	P-Value <0.0001 ****	P-Value <0.0001 ****	P-Value =0.1752 ns	P-Value <0.0001 ****	P-Value =0.0005 ***	P-Value >0.9999 ns	P-Value <0.0001 ****	P-Value >0.9999 ns	P-Value >0.9999 ns
**0.0 µg/mL (Control) vs 100.0 µg/mL**	P-Value <0.0001 ****	P-Value =0.1476 ns	P-Value >0.9999 ns	P-Value <0.0001 ****	P-Value <0.0001 ****	P-Value >0.9999 ns	P-Value <0.0001 ****	P-Value <0.0001 ****	P-Value <0.0001 ****	P-Value <0.0001 ****	P-Value <0.0001 ****	P-Value =0.7993 ns	P-Value <0.0001 ****	P-Value <0.0001 ****	P-Value =0.5621 ns	P-Value <0.0001 ****	P-Value =0.2431 ns	P-Value >0.9999 ns
**0.0 µg/mL (Control) vs 1000.0 µg/mL**	N/A	N/A	N/A	N/A	N/A	N/A	N/A	N/A	N/A	N/A	N/A	N/A	N/A	N/A	N/A	P-Value <0.0001 ****	P-Value <0.0001 ****	P-Value =0.5792 ns
**Day 5**																		
**0.0 µg/mL (Control) vs 0.1 µg/mL**	P-Value =0.0001 ***	P-Value =0.1270 ns	P-Value >0.9999 ns	P-Value <0.0001 ****	P-Value =0.0331 *	P-Value >0.9999 ns	P-Value =0.0001 ***	P-Value <0.0001 ****	P-Value =0.0025 **	P-Value <0.0001 ****	P-Value =0.1895 ns	P-Value =0.8355 ns	P-Value =0.0578 ns	P-Value =0.7131 ns	P-Value >0.9999 ns	P-Value =0.0149 *	P-Value >0.9999 ns	P-Value >0.9999 ns
**0.0 µg/mL (Control) vs 1.0 µg/mL**	P-Value <0.0001 ****	P-Value =0.0034 **	P-Value =0.6026 ns	P-Value <0.0001 ****	P-Value <0.0001 ****	P-Value >0.9999 ns	P-Value <0.0001 ****	P-Value <0.0001 ****	P-Value <0.0001 ****	P-Value <0.0001 ****	P-Value =0.0004 ***	P-Value =0.8190 ns	P-Value <0.0001 ****	P-Value =0.0016 **	P-Value >0.9999 ns	P-Value <0.0001 ****	P-Value =0.9954 ns	P-Value >0.9999 ns
**0.0 µg/mL (Control) vs 10.0 µg/mL**	P-Value <0.0001 ****	P-Value =0.0004 ***	P-Value =0.5562 ns	P-Value <0.0001 ****	P-Value <0.0001 ****	P-Value =0.9940 ns	P-Value <0.0001 ****	P-Value <0.0001 ****	P-Value <0.0001 ****	P-Value <0.0001 ****	P-Value <0.0001 ****	P-Value =0.6318 ns	P-Value <0.0001 ****	P-Value <0.0001 ****	P-Value =0.4441 ns	P-Value <0.0001 ****	P-Value =0.7824 ns	P-Value >0.9999 ns
**0.0 µg/mL (Control) vs 100.0 µg/mL**	P-Value <0.0001 ****	P-Value <0.0001 ****	P-Value =0.0090 **	P-Value <0.0001 ****	P-Value <0.0001 ****	P-Value =0.9185 ns	P-Value <0.0001 ****	P-Value <0.0001 ****	P-Value <0.0001 ****	P-Value <0.0001 ****	P-Value <0.0001 ****	P-Value =0.1571 ns	P-Value <0.0001 ****	P-Value <0.0001 ****	P-Value =0.0017 **	P-Value <0.0001 ****	P-Value =0.0010 **	P-Value >0.9999 ns
**0.0 µg/mL (Control) vs 1000.0 µg/mL**	N/A	N/A	N/A	N/A	N/A	N/A	N/A	N/A	N/A	N/A	N/A	N/A	N/A	N/A	N/A	P-Value <0.0001 ****	P-Value <0.0001 ****	P-Value =0.5768 ns
**Day 6**																		
**0.0 µg/mL (Control) vs 0.1 µg/mL**	P-Value <0.0001 ****	P-Value <0.0001 ****	P-Value =0.5752 ns	P-Value <0.0001 ****	P-Value <0.0001 ****	P-Value =0.7738 ns	P-Value =0.0001 ***	P-Value <0.0001 ****	P-Value <0.0001 ****	P-Value <0.0001 ****	P-Value =0.0007 ***	P-Value =0.3351 ns	P-Value <0.0001 ****	P-Value =0.1058 ns	P-Value =0.9693 ns	P-Value =0.0172 *	P-Value =0.9458 ns	P-Value >0.9999 ns
**0.0 µg/mL (Control) vs 1.0 µg/mL**	P-Value <0.0001 ****	P-Value <0.0001 ****	P-Value =0.1496 ns	P-Value <0.0001 ****	P-Value <0.0001 ****	P-Value <0.0001 ****	P-Value <0.0001 ****	P-Value <0.0001 ****	P-Value <0.0001 ****	P-Value <0.0001 ****	P-Value <0.0001 ****	P-Value =0.0289 *	P-Value <0.0001 ****	P-Value =0.0029 **	P-Value =0.9687 ns	P-Value <0.0001 ****	P-Value <0.0001 ****	P-Value =0.6944 ns
**0.0 µg/mL (Control) vs 10.0 µg/mL**	P-Value <0.0001 ****	P-Value <0.0001 ****	P-Value <0.0001 ****	P-Value <0.0001 ****	P-Value <0.0001 ****	P-Value <0.0001 ****	P-Value <0.0001 ****	P-Value <0.0001 ****	P-Value <0.0001 ****	P-Value <0.0001 ****	P-Value <0.0001 ****	P-Value <0.0001 ****	P-Value <0.0001 ****	P-Value <0.0001 ****	P-Value =0.0677 ns	P-Value <0.0001 ****	P-Value =0.0011 **	P-Value =0.7913 ns
**0.0 µg/mL (Control) vs 100.0 µg/mL**	P-Value <0.0001 ****	P-Value <0.0001 ****	P-Value <0.0001 ****	P-Value <0.0001 ****	P-Value <0.0001 ****	P-Value <0.0001 ****	P-Value <0.0001 ****	P-Value <0.0001 ****	P-Value <0.0001 ****	P-Value <0.0001 ****	P-Value <0.0001 ****	P-Value <0.0001 ****	P-Value <0.0001 ****	P-Value <0.0001 ****	P-Value <0.0001 ****	P-Value <0.0001 ****	P-Value <0.0001 ****	P-Value =0.1120 ns
**0.0 µg/mL (Control) vs 1000.0 µg/mL**	N/A	N/A	N/A	N/A	N/A	N/A	N/A	N/A	N/A	N/A	N/A	N/A	N/A	N/A	N/A	P-Value <0.0001 ****	P-Value <0.0001 ****	P-Value <0.0001 ****
**Day 7**																		
**0.0 µg/mL (Control) vs 0.1 µg/mL**	P-Value <0.0001 ****	P-Value <0.0001 ****	P-Value =0.0024 **	P-Value <0.0001 ****	P-Value <0.0001 ****	P-Value =0.0091 **	P-Value =0.0001 ***	P-Value <0.0001 ****	P-Value <0.0001 ****	P-Value <0.0001 ****	P-Value <0.0001 ****	P-Value =0.0311 *	P-Value <0.0001 ****	P-Value <0.0001 ****	P-Value =0.7671 ns	P-Value =0.0102 *	P-Value =0.9480 ns	P-Value >0.9999 ns
**0.0 µg/mL (Control) vs 1.0 µg/mL**	P-Value <0.0001 ****	P-Value <0.0001 ****	P-Value <0.0001 ****	P-Value <0.0001 ****	P-Value <0.0001 ****	P-Value <0.0001 ****	P-Value <0.0001 ****	P-Value <0.0001 ****	P-Value <0.0001 ****	P-Value <0.0001 ****	P-Value <0.0001 ****	P-Value =0.0021 **	P-Value <0.0001 ****	P-Value <0.0001 ****	P-Value =0.0006 ***	P-Value <0.0001 ****	P-Value <0.0001 ****	P-Value =0.7086 ns
**0.0 µg/mL (Control) vs 10.0 µg/mL**	P-Value <0.0001 ****	P-Value <0.0001 ****	P-Value <0.0001 ****	P-Value <0.0001 ****	P-Value <0.0001 ****	P-Value <0.0001 ****	P-Value <0.0001 ****	P-Value <0.0001 ****	P-Value <0.0001 ****	P-Value <0.0001 ****	P-Value <0.0001 ****	P-Value <0.0001 ****	P-Value <0.0001 ****	P-Value <0.0001 ****	P-Value <0.0001 ****	P-Value <0.0001 ****	P-Value =0.0002 ***	P-Value =0.7161 ns
**0.0 µg/mL (Control) vs 100.0 µg/mL**	P-Value <0.0001 ****	P-Value <0.0001 ****	P-Value <0.0001 ****	P-Value <0.0001 ****	P-Value <0.0001 ****	P-Value <0.0001 ****	P-Value <0.0001 ****	P-Value <0.0001 ****	P-Value <0.0001 ****	P-Value <0.0001 ****	P-Value <0.0001 ****	P-Value <0.0001 ****	P-Value <0.0001 ****	P-Value <0.0001 ****	P-Value <0.0001 ****	P-Value <0.0001 ****	P-Value <0.0001 ****	P-Value =0.0533 ns
**0.0 µg/mL (Control) vs 1000.0 µg/mL**	N/A	N/A	N/A	N/A	N/A	N/A	N/A	N/A	N/A	N/A	N/A	N/A	N/A	N/A	N/A	P-Value <0.0001 ****	P-Value <0.0001 ****	P-Value <0.0001 ****
